# Effects of Sulphate‐Reducing Bacteria Mixed‐Species Biofilms on Microbiologically Influenced Corrosion

**DOI:** 10.1111/1462-2920.70116

**Published:** 2025-08-20

**Authors:** Liam Jones, Maria Salta, Torben Lund Skovhus, Kathryn Thomas, Timothy Illson, Julian Wharton, Jeremy Webb

**Affiliations:** ^1^ School of Biological Sciences University of Southampton Southampton UK; ^2^ Endures, MIC and Biofilm Department Den Helder the Netherlands; ^3^ Research Centre for Built Environment, Climate and Water Technology VIA University College Horsens Denmark; ^4^ DNV Loughborough UK; ^5^ School of Engineering University of Southampton Southampton UK; ^6^ National Biofilms Innovation Centre Southampton UK

**Keywords:** biofilms, carbon steel, electroactive microorganisms, microbiologically influenced corrosion, multiple lines of evidence, standardisation

## Abstract

Sulphate reducing prokaryotes are widely acknowledged as key contributors to microbiologically influenced corrosion in industry. Characterisation of their behaviour within mixed‐species biofilms that reflect ecologically relevant conditions is limited. A novel dual anaerobic biofilm reactor protocol allowed a complex microbial consortium to be investigated. Continual biofilm growth resulted in significantly greater corrosive pit density, with 15 and 47 pits mm^−2^ in the biotic reactor for as received (AR) and polished (P) coupons respectively. There was an average pit density of 3 pits mm^−2^ in the abiotic reactor for both AR and P coupons. Moreover, a greater pit depth and size were observed when compared to the sterile abiotic conditions. Identifying and understanding the relative contributions of different microbial mechanisms within mixed‐species biofilms is critical. Importantly, electroactive and corrosive *Desulfovibrio desulfuricans* and *Desulfovibrio vulgaris* were identified within the biofilm. These microorganisms play a crucial role in extracellular electron transfer, a key process in microbiologically influenced corrosion. The protocol not only deepens the mechanistic understanding of MIC but also offers a versatile tool for testing mitigation strategies under realistic and customizable conditions. This integrated approach can ultimately support the development of more targeted, sustainable corrosion prevention and management practices.

## Graphical abstract

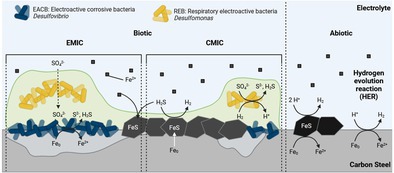

## 1 Introduction

Sulphate reducing prokaryotes (SRP), particularly sulphate‐reducing bacteria (SRB), are ubiquitous anaerobes and among the most studied microorganisms in microbiologically influenced corrosion (MIC) (Hamilton 1985; Lee et al. 1995). SRB respire sulphate (${\mathrm{SO}}_{4}^{2-}$), reducing it to sulphide species ($H_{2}S$, ${\mathrm{HS}}^{-}$), using organic compounds like lactate as electron donors, which are oxidised to CO_2_ or partially to acetate (Venzlaff et al. 2013; Sorokin 1966). Many SRB can also utilise hydrogen ($H_{2}$), a common by‐product in anoxic environments such as marine sediments (Venzlaff et al. 2013). These systems often host mixed‐species biofilms, where microbial interactions, mutualistic or competitive, shape nutrient availability through metabolic activity and microscale gradients. Corrosion product formation may further influence local redox conditions by generating electrochemical cells that modulate anodic and cathodic reactions. Understanding how such biofilms and their by‐products affect community dynamics and corrosion mechanisms is essential, particularly in marine and energy infrastructure contexts.

The mechanisms by which SRB interact with metallic ${\mathrm{Fe}}^{0}$ have been a topic of debate in the literature, with two prevailing corrosion mechanisms proposed (Costello 1974; Booth and Tiller 1960; Hardy 1983), Electrical Microbial Influenced Corrosion (EMIC) and Chemical Microbial Influenced Corrosion (CMIC) (Venzlaff et al. 2013; Enning and Garrelfs 2014). EMIC is a cathodic reaction, that is kinetically impossible under sterile abiotic conditions, which is characterised by the direct electron uptake of electrons from ${\mathrm{Fe}}^{0}$ oxidation by SRB and the formation of large amounts of inorganic corrosion products (Enning and Garrelfs 2014). Importantly, EMIC can occur either directly or indirectly through extracellular electron transfer (EET). CMIC is fundamentally different and is characterised by the indirect activity of metabolic end products. Through SRB respiration, the biogenic production of $H_{2}S$ initially stimulates the anodic part of the corrosion reaction by chemisorption and direct reaction with metallic ${\mathrm{Fe}}^{0}$ to form inorganic corrosion products. Cathodic reactions become more important drivers of ${\mathrm{Fe}}^{0}$ oxidation once the metallic surface is covered with inorganic corrosion products such as FeS (Newman et al. 1991; Sun and Nešic 2007).

(1) $$H_{2}S+{\mathrm{Fe}}^{0}\to H_{2}+\mathrm{FeS}$$

(2) $$3\left({\mathrm{CH}}_{2}O\right)+2{\mathrm{Fe}}^{0}+{2\mathrm{SO}}_{4}^{2-}+H^{+}\to {2\mathrm{HCO}}_{3}^{-}+2\mathrm{FeS}+2H_{2}O$$

Traditionally, the literature has primarily focused on single‐species studies (Xu and Gu 2014; Jia et al. 2018; Enning et al. 2012). Whilst these types of studies provide clear, simple, and reproducible results; ultimately, they lack ecological relevance and do not consider microbial interactions within established microbiomes. However, distinguishing between abiotic and biotic corrosion mechanisms is simpler in these types of studies. Moreover, identifying key electron acceptors and donators is less complex. It was speculated by early investigators that a $H_{2}$ film develops at the metal surface in the absence of microorganisms leading to polarisation and impeding ${\mathrm{Fe}}^{0}$ dissolution (Costello 1974). Yet, in the presence of microorganisms with the capability of $H_{2}$ utilisation, it was suggested that through the hydrogen evolution reaction (HER) these microorganisms would lead to depolarisation, allowing ${\mathrm{Fe}}^{0}$ dissolution to proceed (Venzlaff et al. 2013). In turn, this makes the metal surface more reactive, thus accelerating the overall corrosion process. This was known as the ‘cathodic depolarisation theory’ (Venzlaff et al. 2013). However, whilst this reaction can proceed spontaneously from a thermodynamic perspective, early investigators did not consider environmentally relevant conditions or account for kinetic considerations (Venzlaff et al. 2013).

Several cultures of SRB were shown to stimulate the cathodic current on mild steel electrodes in early studies (Booth and Tiller 1960; Booth and Tiller 1962). The authors attributed this to bacterial $H_{2}$ uptake from the electrode surface and, interpreted the observation in favour of the ‘classical’ depolarisation theory (Venzlaff et al. 2013). However, an experimental misconception in the early electrochemical study of the postulated direct mechanism involving $H_{2}$ with conventional SRB strains was the addition of lactate, which represents a competitive electron donor in addition to ‘cathodic’ $H_{2}$ (Venzlaff et al. 2013; Booth and Tiller 1960; Booth and Tiller 1962). More importantly, lactate leads to excessive concentrations of aggressive hydrogen sulphide ($H_{2}S$) which cause chemical corrosion (Venzlaff et al. 2013). Studies which omitted lactate showed that SRB became more corrosive even though such starvation led to reduced sessile cell densities on metallic ${\mathrm{Fe}}^{0}$ (Xu and Gu 2014). Additionally, cathodic depolarisation was shown not to occur in SRB cultures with metallic ${\mathrm{Fe}}^{0}$ as the only source of electrons for the organisms (Costello 1974; Hardy 1983). This was because SRB biofilms switched to elemental ${\mathrm{Fe}}^{0}$ as a substitute for organic carbon as an electron donor in their respiration (Jia et al. 2018). Conversely, acceleration of the cathodic reaction was shown to result from the reactivity of dissolved $H_{2}S$ (Venzlaff et al. 2013). In another approach towards a mechanistic understanding of anaerobic corrosion, SRB were directly enriched and isolated with metallic ${\mathrm{Fe}}^{0}$ as the only source of electrons, without an organic substrate such as lactate (Venzlaff et al. 2013; Dinh et al. 2004). In these studies, the SRB severely corroded the metallic surface with a rate of up to 0.7 mm year^−1^. This corrosion rate (*CR*) could not be explained by dependency on H_2_ alone (Dinh et al. 2004). Their findings indicated a direct electron uptake through EMIC.

The biocatalytic cathodic sulphate reduction (BCSR) theory describes how SRB directly influence MIC by coupling ${\mathrm{SO}}_{4}^{2-}$ to $H_{2}S$, ${\mathrm{HS}}^{-}$, with electron uptake under anaerobic conditions (Xu et al. 2013). These sulphide ions react with ${\mathrm{Fe}}^{2+}$ to form FeS, which may facilitate electrochemical cell formation, altering anodic and cathodic reactions and thus impacting corrosion rates (Jia et al. 2018). $H_{2}S$ concentrations are typically higher under SRB biofilms than in the bulk fluid due to cell density and diffusion limitation. While SRB‐driven corrosion is significant, other abiotic and biotic processes also contribute in mixed‐species biofilms. Though ecologically relevant, such systems introduce complexity that can obscure mechanistic interpretation.

This study aimed to demonstrate the applicability and reproducibility of a novel dual bioreactor protocol to investigate the influence of a mixed‐species SRB biofilm on carbon steel (UNS G10180 CS) corrosion. Unlike conventional studies that often rely on pure cultures or simplified microbial consortia, our protocol uses a marine sediment‐derived, ecologically relevant mixed‐species consortium under continuous flow anaerobic conditions. This system mimics natural biofilm formation more closely and allows for time‐resolved monitoring of corrosion mechanisms under evolving environmental and microbial conditions. A key focus was placed on enumerating SRP using selective ATCC 1249 Modified Baar's (MB) media to evaluate the influence of a mixed‐species biofilm on the prevailing corrosion mechanisms. Furthermore, the integration of multiple lines of evidence (MLOE) (Knisz et al. 2023), incorporating microscopy‐based methods, microbiological molecular methods (MMM), and electrochemical techniques offers a holistic assessment of both microbial community dynamics and corrosion behaviour. This multidisciplinary, systems‐level approach is a key advance over existing MIC protocols.

## 2 Materials and Methods

### 2.1 Test Conditions

Two anaerobic CDC (Center for Disease Control) biofilm reactors (Biosurface Technologies Corporation) were used: an abiotic control reactor and a biotic test reactor (key dimensions: 22 cm reactor height and 12 cm internal diameter; 21 cm coupon holder rod; 1.27 cm coupon diameter). Sterile carbon steel coupons were fixed in reactors and exposed to two separate conditions for 28 days. Anaerobic conditions were maintained throughout the test by initially sparging the system with nitrogen gas (oxygen free nitrogen) (BOC Nitrogen (Oxygen Free), 44‐W) over an initial three‐day batch phase. Anaerobic conditions, considered to be hypoxic or low $O_{2}$, are characterised as a system with low concentrations ranging between 1% and 30% saturation. Strict obligate anaerobes will not survive if there is more than half a percent $O_{2}$ in the environment, while moderate obligate anaerobes can still grow in a 2% to 8% $O_{2}$ environment (Rabalais et al. 1999). ATCC 1249 Modified Baar's (MB) media with resazurin solution (0.1%, 0.5 mL L^−1^) (Merck) was used as the growth medium. The test media composition can be seen in Table S1. It was acknowledged that yeast extract contains redox mediators that may adsorb onto the electrode surfaces and chelate metal ions and the test matrix was designed to highlight any significant interference (Knisz et al. 2023; Lee and Little 2015). Resazurin was added as a redox indicator, as it is colourless under oxygen free conditions but changes to a pink colour in an oxygen‐containing environment. Agitation of the reactor baffles was set to 50 rpm to maintain a homogeneous solution. The reactor temperature was at ambient conditions (20°C). Prior to inoculating the biotic reactor, a three‐day pre‐culture was prepared in a blue‐cap flask (50 mL), consisting of 10% marine sediment with the remainder fresh MB media. The biotic reactor was inoculated using a sterile syringe, where 10% of the working reactor volume (35 mL) was added as the inoculum. Initial adenosine triphosphate (ATP) measurements were taken from the pre‐culture and long‐term frozen stocks were prepared using 20% glycerol. Figure S1a shows a schematic of the full experimental setup, with Figure S1b illustrating the three‐electrode cell setup within each anaerobic CDC biofilm reactor. Both reactors were operated in batch mode for the first 3 days to allow settlement and to facilitate biofilm formation in the biotic reactor. After this period, the reactors were switched to continuous flow of fresh media at a rate of 0.2 mL min^−1^, which replaced about 50% of the 600 mL total volume daily (288 mL day^−1^).

### 2.2 Microbial Consortia

The sheltered zone littoral sediment microbial consortia were collected at a depth between 10 and 15 cm below the sediment surface during low tide from Langstone Harbour, United Kingdom (50°50′11.9″N 0°58′47.5″W). The coastal/estuarine marine sediment (very fine and cohesive mud and silt deposits) was selected to sample microorganisms living under low oxygen conditions. The environmental conditions on the day the marine sediment was collected are presented in Table S2. The sediment samples were added to 500 mL of the MB medium and stored at 37°C in an anaerobic chamber to maximise the recovery of the diverse microbial populations. Mesophilic bacteria can survive and grow in temperatures between 20°C and 45°C (Schiraldi and De Rosa 2014). Thus, a tropic strategy to promote cell growth and viability was employed to maximise microbial recovery. The anaerobic chamber gas mixture consisted of 85% N_2_, 10% CO_2_, and 5% H_2_ (BOC Anaerobic Growth Mix, 290,563‐L). Long‐term storage of sediment samples and microbial consortia was employed to create frozen stocks at −80°C.

### 2.3 Carbon Steel Coupon Preparation

UNS G10180 (AISI 1018) carbon steel disc coupons (Biosurface Technologies—RD128 CS), with dimensions of 12.7 mm diameter × 3.8 mm thickness, were used in either the as‐received (AR) (*R*_a_ = 1.14017 ± 0.21778) condition or polished (P) (*R*_a_ = 0.43175 ± 0.03615) with a Kemet 15 Lapping machine using 25 μm Type K diamond slurry. The surface profiles and weights for all coupon samples were assessed prior to starting the experiment, on Day 0, for surface profilometry and gravimetric analysis to be performed at the completion of the experiment after Day 28. Three‐dimensional (3D) surface profiles were taken using a 3D optical profilometer (Alicona imaging infinite focus microscope IFM G4 3.5). A Mettler AT201 was used to take five measurements of the initial weights of all coupons.

### 2.4 Experimental Setup

Before autoclaving, the two anaerobic CDC biofilm reactors were cleaned with detergent and allowed to dry. The empty reactors with attached tubing were placed in autoclavable bags; all tube openings and air filters (Millex, 0.2 μm) were covered in aluminium foil, with tube openings clamped shut. The empty assembled reactors were autoclaved for 15 min at 121°C, along with prepared MB test media. After cooling, the reactors were transferred into a sterilised microbiological safety cabinet, along with all rods, carbon steel test coupons, as well as any sensors and electrodes. Working electrode rods were prepared in advance. For each working electrode rod, wires were soldered to each coupon separately. The coupon face with the soldered wire was then covered with a lacquer solution (Polishing Shop, Type 45 Stop Off Lacquer) and allowed to dry. To assemble the reactors, all rods with coupons were submerged in 99% ethanol for at least 10 s, then inserted into the autoclaved reactors. Any sensors or electrodes used in place of a rod were also inserted, after also being sterilised with 99% ethanol for at least 10 s. The medium bottles and all tubing were connected in a microbiological safety cabinet. Once both reactors were fully assembled, they were transferred to the working area, with access to a N_2_ gas supply. The tubing was evenly split into each reactor to equalise the pressure gradient caused by the peristaltic pump (Matson Marlow 300 series).

### 2.5 Sulphide Analysis

Sulphide concentrations were monitored daily in each reactor using a Unisense, SULF‐50 sulphide microsensor (50 μm diameter) and amplifier (Unisense, $H_{2}S$ UNIAMP). The microsensor measures the partial pressure of $H_{2}S$ gas, and the total concentration is a function of pH and temperature. The microsensor limit of detection is 0.3 μM, with a range from 0 to 300 μM sulphide in water. Calibration utilised the $H_{2}S$ and SULF sensor calibration kit (Unisense, CALKIT‐$H_{2}S$). Due to the nature of the experimental setup, it was not possible to calibrate the microsensors during the experiment. However, calibrations were performed both prior to starting the experiment and once the experiment had finished to confirm that the sensors were still calibrated. The SensorTrace Suite software was used to collect the sulphide microsensor data. The sensor has a higher signal for zero right after it has been connected to the amplifier, thus each microsensor collected readings for 5 min (approximately 300 data points) on each day. This was to allow the sensor to stabilise.

### 2.6 Surface Profilometry and Visual Inspection

Corrosion products and biofilms were removed from the surface using the cleaning protocol as described below for the gravimetric analysis. Three‐dimensional (3D) profiling of the carbon steel surfaces was reconstructed using an Alicona imaging infinite focus microscope IFM G4 3.5. The images allowed assessment of changes in surface roughness compared to the surface profiles obtained prior to testing. Additionally, ImageJ/Fiji was used for the quantitative determination of pit depth, width, height, percentage area, and to assess pit rate (*PR*) and pit density (*PD*). This analysis was performed on 15 total locations on three coupons (five locations each) for both the AR and P coupons. The method involved applying a colour threshold to depths greater than 5 μm. Then, the images were converted to a binary mask. Next, measurement parameters were selected for areas greater than 650 μm^2^. Finally, the images were analysed to display counts, area, and average size of pits. The pit parameters were adapted from ASTM G48‐11 (ASTM G48‐11 2020). For pit rate analysis, the deepest pits from each image were captured using the Alicona. Pit rates were calculated using the formula described in NACE SP0775‐2023 (NACE SP0775‐2023 2023).

### 2.7 Gravimetric Analysis

Corrosion products and biofilms were removed following the ASTM G1‐03 standard with a 15% inhibited hydrochloric acid described in NACE SP0775‐2023 (ASTM G1‐03 2018; NACE SP0775‐2023 2023a). A stock solution was made of 37.5% HCl (Merck, Suprapur, 1.00318.0500) to which 10 g/L of 1,3‐di‐n‐butyl‐2‐thiourea (DBT) (Merck, 8.20423.0250) was added. Immediately prior to use, the stock solution was diluted by slowly adding a measured volume of stock solution to an equal volume of deionised water with stirring. A Mettler AT201 was used to take five measurements of all coupons. Corrosion rates were determined by the gravimetric technique that considers the weight loss and surface area of the metal samples described in NACE SP0775‐2023 (ASTM G48‐11 2020).

### 2.8 Electrochemical Analysis

Electrochemical measurements were performed using a Gamry Instruments potentiostat (Ref 600 Plus). The electrochemical behaviours of the carbon steel coupons were evaluated using a three‐electrode system consisting of a UNS G10180 coupon as the working electrode, a graphite rod (Alfa Aesar, 99.9995%, 6.15 mm diameter, 152 mm long) as the counter electrode, and a silver/silver chloride (Ag/AgCl, 3.5 M KCl) reference electrode (Sentek, (AgCl) Double junction Reference Electrode). On day 1, after the test reactor was inoculated, both reactors were left for at least 1 h prior to performing any electrochemical measurements. Open‐circuit potentials (OCP) were recorded for each coupon on day 1 prior to measuring linear polarisation resistance (LPR) and electrochemical impedance spectroscopy (EIS). LPR and EIS were measured daily for each sample. LPR measurements were performed from ±10 mV with respect to *E*_OCP_ using a scan rate of 0.167 mV s^−1^. EIS measurements were performed at OCP with an applied 10 m*V*_rms_ sinusoidal potential signal with a frequency range of 10^−2^ to 10^5^ Hz. Potentiodynamic polarisation measurements were performed at the end of the experiment on day 28 for each coupon from −0.200 mV to +0.200 V using a scan rate of 0.5 mV s^−1^. Standard procedures were followed when selecting an equivalent circuit best fit using the Gamry Echem Analyst software: (*i*) the chi‐squared (*χ* ^2^) error was suitably minimised (*χ* ^2^ ≤ 10^−4^) and (*ii*) the errors associated with each element were ranged between 0% and 5%.

### 2.9 Confocal Laser Scanning Microscopy and Post‐Image Analysis

The distribution of live and dead cells within biofilms was studied using confocal laser scanning microscopy (CLSM). Coupons were gently rinsed with sterile anaerobic PBS (pH 7.4), with the following composition: NaCl 8 g, KCl 0.2 g, Na_2_HPO_4_ 1.44 g, KH_2_PO_4_ 0.245 g, deionised water 1 L, and stained using the FilmTracer Live/Dead biofilm viability kit (Invitrogen) according to the manufacturer's instructions. Before imaging with a Leica SP8 confocal microscope, coupons were rinsed with sterile deionised water to remove the excess of dyes and fixed using mowiol. Mowiol had the following composition: 2.4 g Mowiol, 6 mL deionised water, 12 mL 0.2 M Tris (pH 8.5), 0.01 g sodium azide, and 6 g glycerol. Images were obtained with a 63× magnification and glycerol immersion. The dyes used stained live cells with a green‐fluorescent colour (SYTO 9) and dead cells with a red colour (propidium iodide). The *z*‐stacked images were analysed using Imaris software (Oxford Instruments).

### 2.10 Microbial Community Analysis

After 28 days, three AR and three P coupons were gently rinsed with PBS and then placed in a Falcon tube containing 10 mL of MB solution. Long‐term frozen stocks were prepared using 20% glycerol for the bulk fluid, AR biofilm, and P biofilm samples from the biotic reactor. The sediment, three‐day pre‐culture, day 28 bulk fluid, AR biofilm, and P biofilm frozen stocks were sent in triplicate for DNA extraction and 16S rRNA amplicon sequencing. PCR amplification was completed with primer pairs 347F and 800R. Library preparation and sequencing were performed for the V3 and V4 regions of the 16S rRNA gene targeting both Bacteria and Archaea. The microbiome analysis pipeline, along with DNA extraction, was performed by Eurofins Genomics LLC. The Illumina platform was used, sequencing on MiSeq with the 2 × 300 bp paired‐end read module. Taxonomic classification method used Kraken2 (v 2.1.1). Bioinformatics and data analysis was performed using the Qiime2 (version 2023.5) software. To visualise the multivariate dispersion of the community composition, a principal component analysis (PCA) analysis was conducted employing GraphPad (version 10.0.2).

### 2.11 ATP Assay

The ATP concentration in both the abiotic and biotic reactors was determined by luminescence after reaction with luciferin‐luciferase using the BacTiter‐Glo Microbial Cell Viability Assay kit (Promega). The assay provides a method for determining the number of viable microbial cells in culture based on quantification of the ATP present. ATP is the energy source of all living cells and is involved in many vital biochemical reactions. When cells die, they stop synthesising ATP and the existing ATP pool is quickly degraded. Higher ATP concentration indicates a higher number of living cells. All assays were performed according to the manufacturer's instructions. Six coupons, three AR and three P, were gently rinsed with PBS and then immersed in a Falcon tube containing 10 mL of MB solution. Any cells were detached from the metal coupons using a cell scraper (Biologix). Both planktonic and sessile samples were processed with the BacTiter‐Glo Microbial Cell Viability Assay kit, which measures ATP from as few as 10 microbial cells. The ATP concentrations were determined by measuring luminescence with a Clariostar Plus Multimode Microplate Reader (BMG Labtech). Planktonic cells in each reactor were determined following the same method described before; in this case, 10 mL of the bulk test solution was processed with the BacTiter‐Glo Microbial Cell Viability Assay kit. Negative controls of PBS, deionised water and MB solution were used to indicate no ATP activity.

### 2.12 Corrosion Product Analysis

Note, for this study no chemical analysis was performed on the corrosion products due to access to the XRD being unavailable as it was out of order. Thus, the hypothesised presence of FeS for this study could not be confirmed and was inferred from the literature (Al‐Abbs, Williamson, et al. 2013; Al‐Abbs, Bhola, et al. 2013; Al‐Abbs, Williamson, et al. 2013a).

## 3 Results

### 3.1 Visual Observations

Throughout the 28 days of experimentation, the abiotic media had no visual changes, and the CS coupons maintained their silver‐grey metallic lustre appearance. The sterile abiotic reactor MB media was orange/pink in colouration with no apparent turbidity. However, after inoculation of the biotic reactor MB media, a black surface film was evident on the steel coupons (day 1), with a low level of turbidity. After the flow of fresh MB media was started on day 4, over the following days up to day 7, black particulates in the MB media started to precipitate at the reactor bottom, with the bulk media being green in appearance. After 2 weeks, there was a visible surface crust at the MB interface, and the media was black in appearance with high turbidity. Over the following 2 weeks, up to 28 days, the biotic media was dark green/black in colouration. Upon dismantling of the reactors on day 28, and retrieval of the coupon rods, there was a significant difference in the coupon appearances; see Figure S2. The abiotic surfaces had no apparent corrosion products, whilst the biotic surfaces were covered with a thick slimy biofilm with a dark green/black granular deposit.

### 3.2 Sulphide Analysis

**FIGURE 1:**
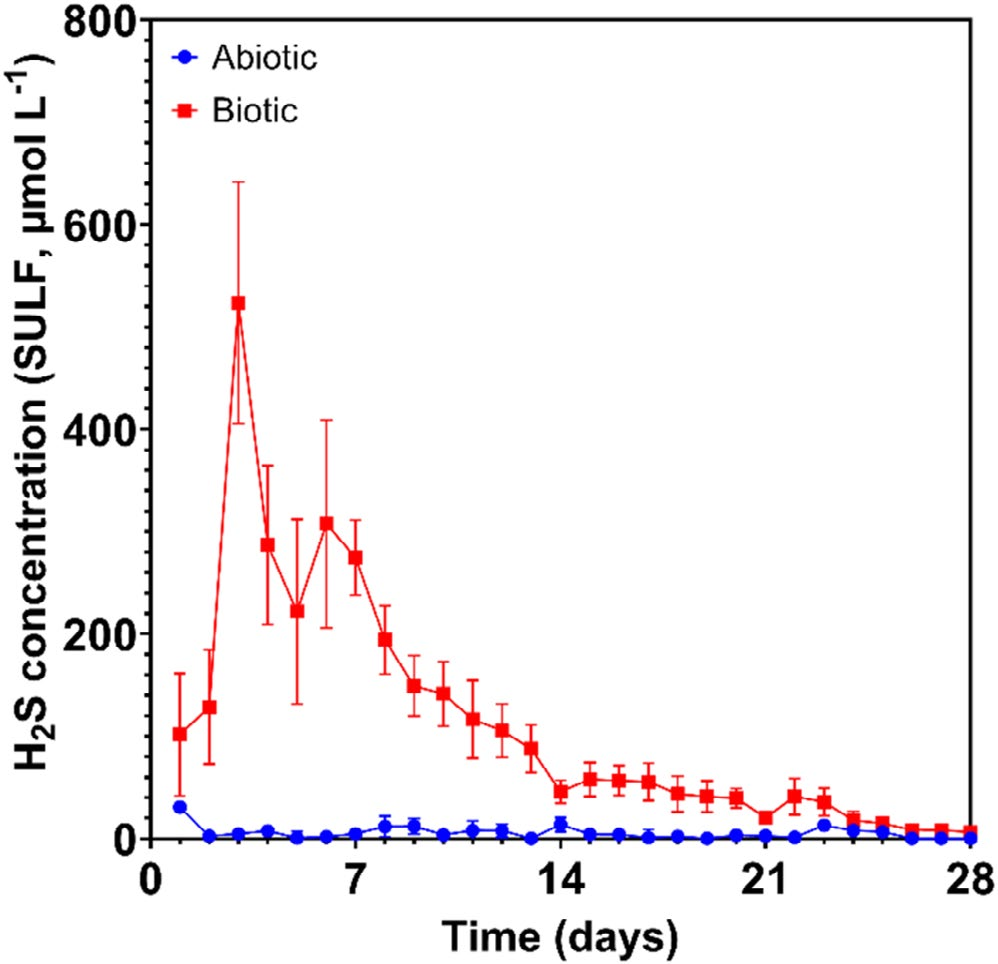
Aqueous sulphide measurements (*SULF*, μmol L^−1^) for the abiotic and biotic conditions over 28 days (nb. measured the anaerobic MB media in situ adjacent to corroding UNS G10180 carbon steel).

Figure 1 shows the aqueous $H_{2}S$ concentrations monitored in the abiotic and the biotic anaerobic MB media over the test duration. For the sterile abiotic condition, there was a generally low $H_{2}S$ concentration (mean: 5.1 μmol L^−1^). Whereas for the biotic condition, the $H_{2}S$ concentration drastically increased after the flow of fresh MB media on day 3, to a maximum of 523.4 μmol L^−1^, before ultimately decreasing to similar levels detected for the abiotic media after 2 to 3 weeks. Dissolved oxygen (DO) concentrations measured on day 28 were: 4.6 ppm in the 10 L media containers, 0.5 ppm in the abiotic and 0.0 ppm (below the limit of detection) in the biotic reactor. The pH was not measured on completion of the experiment. Whilst the media containers were initially sparged with nitrogen gas, minor oxygen ingress was observed over time, due to the pressure differential caused by the constant flow of media.

### 3.3 Carbon Steel Surface Analysis

**FIGURE 2:**
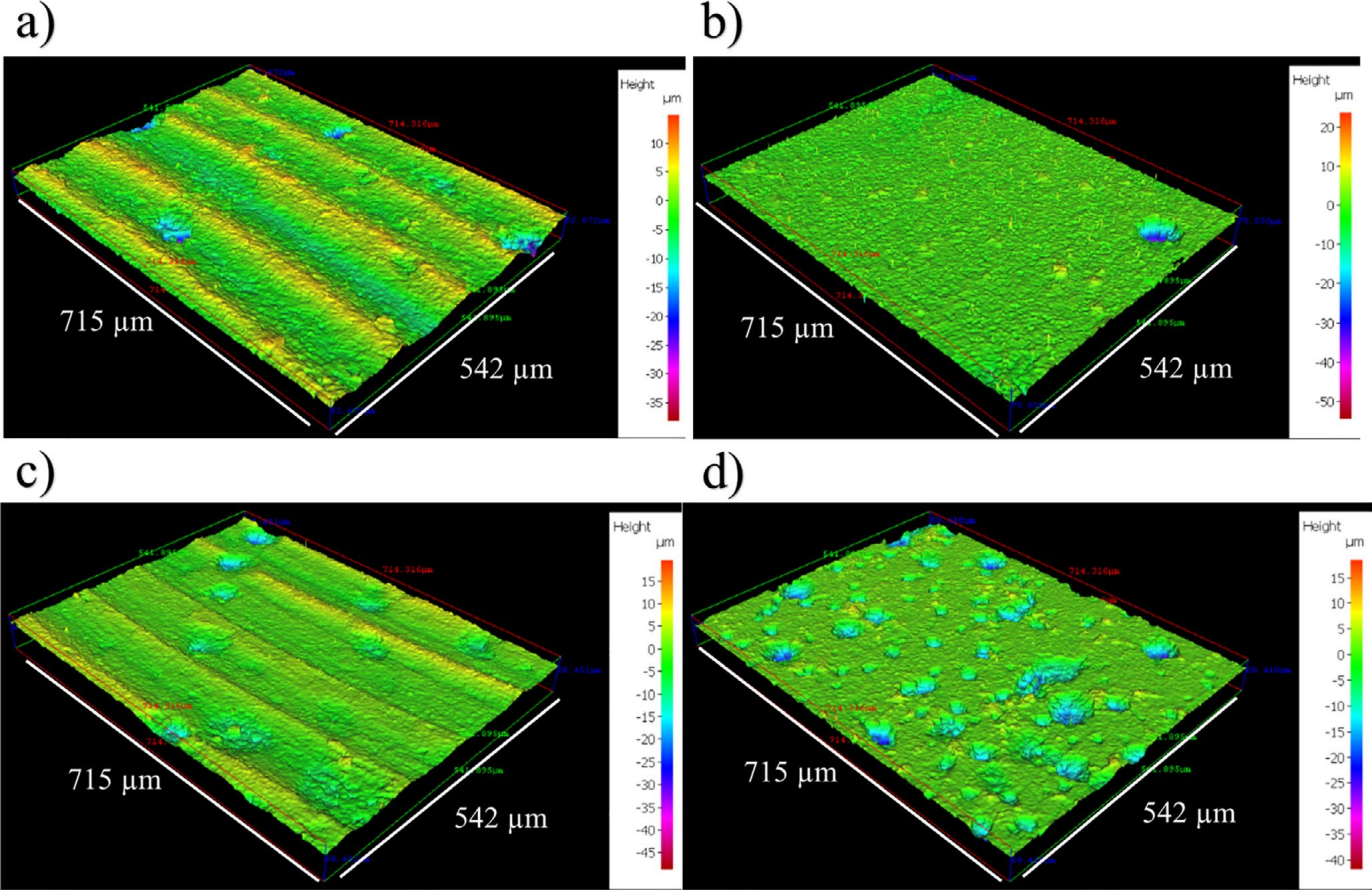
Three‐dimensional optical surface profilometry of the cleaned UNS G10180 surfaces at day 28. AR coupons for: (a) abiotic and (b) biotic conditions; and P coupons for: (c) abiotic and (d) biotic conditions, after exposure to anaerobic MB media for 28 days.

Figure S3 shows the CS surfaces on day 0. Table S3 summarises the quantitative surface roughness profiles on both day 0 and day 28. Figure 2 shows the cleaned CS surfaces after 28 days, with biofilms and corrosion products removed to reveal the morphology of the surface degradation and to facilitate corrosion assessment. Surface profilometry revealed that both abiotic and biotic anaerobic MB media led to localised pitting, with the biotic condition more extensively pitted. Chemical heterogeneity, differential aeration, or surface defects can all contribute to localised pitting. In both abiotic and biotic conditions, these factors interact synergistically, with biotic conditions often amplifying the effects. The abiotic average pit depths were 39 and 31 μm, and average pit areas of 1212 and 1022 μm^2^, for the AR and P coupons, respectively. The biotic average pit depths were 38 and 41 μm, with the average pit areas of 1571 and 1530 μm^2^, for the AR and P coupons, respectively. Again, for this study a pit was classified as having a depth greater than 5 μm and an area greater than 650 μm^2^ (ASTM G48‐11 2020).

**FIGURE 3:**
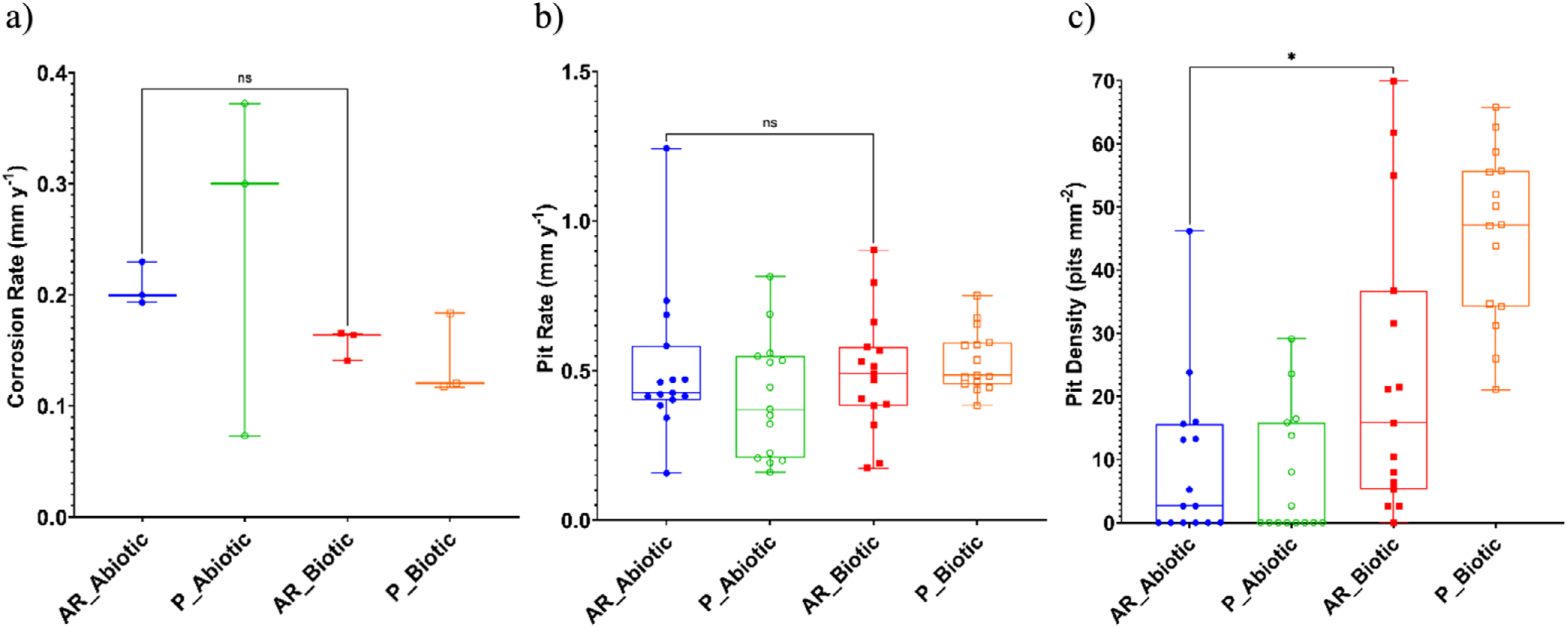
Abiotic and biotic corrosion performance after exposure to anaerobic MB media for 28 days: (a) corrosion rate via gravimetric analysis and surface profilometry assessed (b) pit rate and (c) pit density (*p* < 0.05 for the abiotic and biotic AR coupons, *p* < 0.0001 for the abiotic and biotic P coupons, *p* < 0.0066 for the biotic AR and biotic P coupons), for the AR and P coupons.

Figure 3a provides an evaluation of the CS coupons *CR*. For the abiotic condition, there was no significance in *CR* when compared to the biotic condition. Furthermore, when comparing between the two surface roughness types, AR and P coupons within each reactor, there was also no significance (*n* = 3). According to the NACE SP0775‐2023 assessment criteria, there was a high *CR* (between 0.13 and 0.25 mm year^−1^) in the abiotic and a moderate *CR* (between 0.025 and 0.12 mm year^−1^) in the biotic (Figure 3a); whilst a severe *PR* (> 0.38 mm year^−1^) was assessed for both the abiotic and biotic conditions (Figure 3b) (NACE SP0775‐2023 2023). Further analysis of the surface profilometries in Figure 2 allowed a quantitative determination of the *PR* Figure 3b and *PD* Figure 3c of the CS coupons. For the biotic reactor, though there was a higher *PR*, there was no significant difference evident between the abiotic and biotic conditions, though *PR* is calculated based on the deepest pits. Whilst *PR* had no significant difference, there was a significant increase in the incidence of pitting (*p* < 0.05). There was an average *PD* of 3 pits mm^−2^ in the abiotic reactor for both AR and P coupons, with 15 pits mm^−2^ and 47 pits mm^−2^ in the biotic reactor for AR and P coupons, respectively. Again, there were no significant differences when comparing between the two surface roughness types within each reactor.

### 3.4 Electrochemical Measurements

**FIGURE 4:**
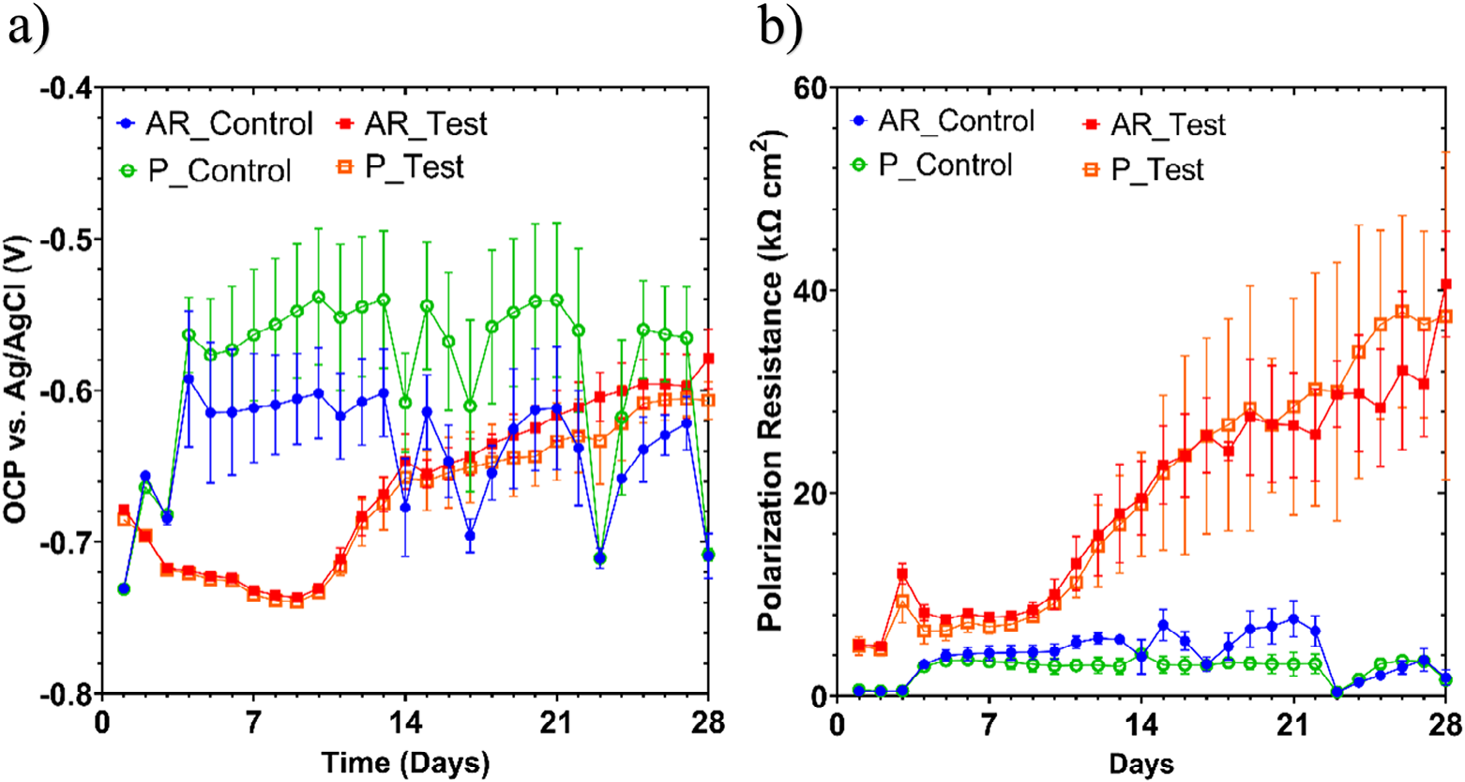
LPR data for UNS G10180 carbon steel: (a) open‐circuit potentials and (b) polarisation resistance in anaerobic MB media (abiotic and biotic conditions), for AR and P coupons (data points represent mean ± standard deviation, *n* = 3). Reactor stirrer at 50 rpm.

**FIGURE 5:**
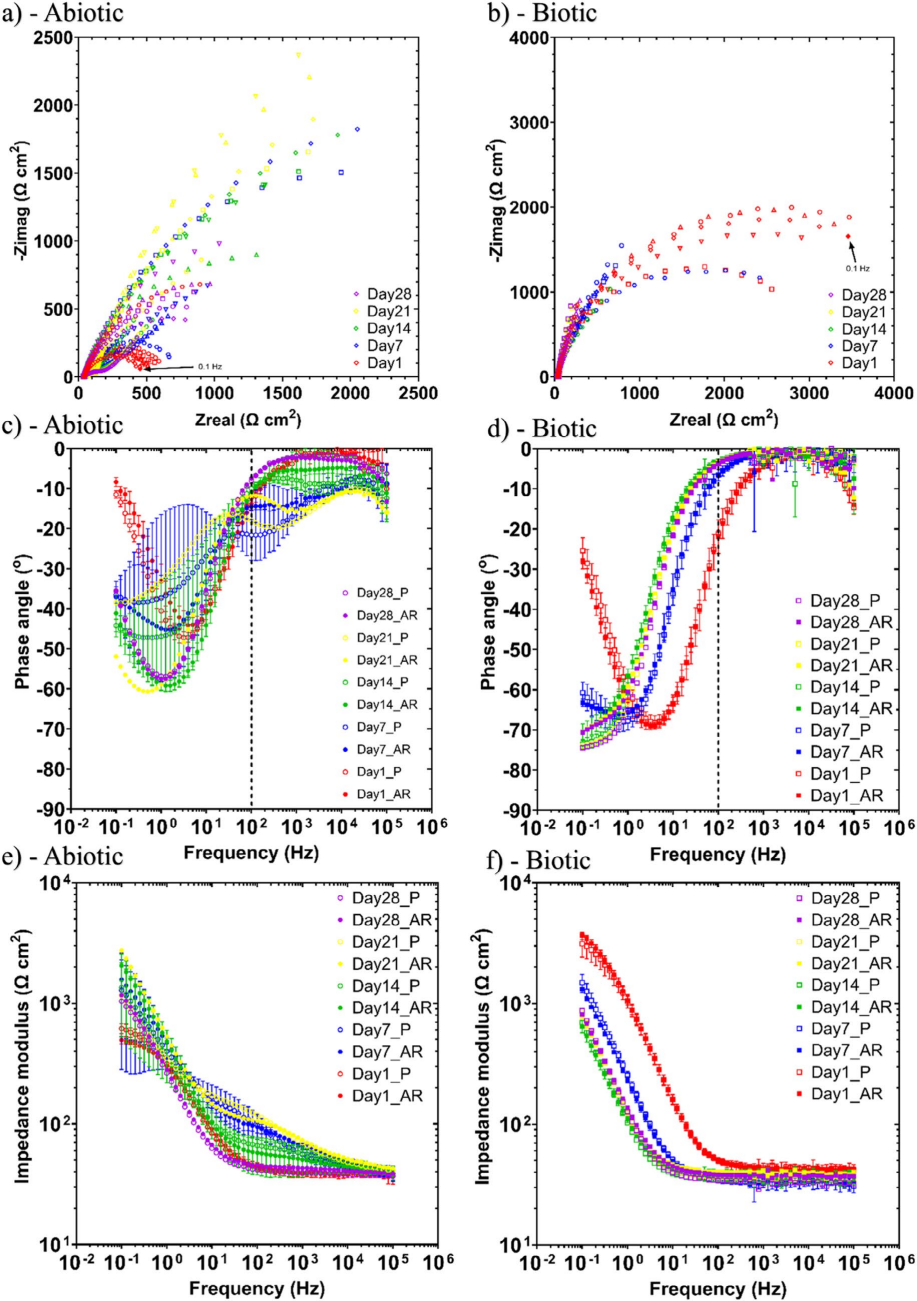
EIS data for UNS G10180 carbon steel in anaerobic MB media at OCP: (a, b) Nyquist, (c, d) Bode phase angle (*θ* vs. *f*), and (e, f) Bode impedance modulus (|*Z*| vs. *f*) over 28‐days. (*n* = 3). Reactor stirrer at 50 rpm.

Figure 4 shows the changes in *E*_corr_ and *R*_p_ between the abiotic and biotic anaerobic MB media, for the UNS G10180 CS coupons. The abiotic condition, Figure 5a, had a distinct +0.100 V increase in the *E*_corr_ during the first 3 days that can be linked with the presence of a conditioning film (i.e., an adsorbed organic layer). Generally, a pseudo‐steady state *E*_corr_ was attained in the days following with some odd electronegative shifts. Conversely, for the biotic condition, there was a gradual electronegative shift in the *E*_corr_ after the first day, until Day 9, after which *E*_corr_ steadily increased by +0.070 V to around −0.610 V on Day 28. The potential for the abiotic condition in the latter stages was generally around −0.610 V versus Ag/AgCl too.

In Figure 4b the LPR derived *R*_p_ remained low at approx. 300 Ω cm^2^ for the sterile abiotic condition. Whereas there was a gradual increase for the biotic condition over the 28 days, with *R*_p_ ranging between 5000 and 40,000 Ω cm^2^. The pioneering bacterial attachment/colonisation, biofilm formation, and growth kinetics will lead inevitably to a more complex electrochemical response. Overall, there were no significant differences when comparing between the two surface roughness's (AR and P) within both the abiotic and biotic reactor environments.

Figure 5 shows the EIS data for UNS G10180 CS in the anaerobic MB media presented in three forms: Nyquist, Bode phase angle, and Bode impedance modulus plots. The sterile abiotic condition on Day 1 has a depressed Nyquist semicircle and phase angle close to zero, which is indicative of a resistive response. However, on the subsequent days up until Day 28, the abiotic condition exhibits a typical electrochemical response for the formation of a porous interface, with diffusion of soluble electroactive species across an organic conditioning film (Flemming et al. 2009). The diffusive behaviour is associated with linear features having a roughly 45° slope (a Warburg impedance response) and phase angles close to 40° in the low frequency region (10^−2^ to 10^0^ Hz), see Figure 5a,c. The abiotic impedance spectra shift towards lower frequencies (10^−2^ to 10^0^ Hz), with a combined diffusive/resistive behaviour. The biotic condition had a consistently uniform EIS response over the 28 day test, with only minor variation in the spectra, and suggests the absence of significant detectable electrochemical changes with time. On Day 1, there is a more capacitive/diffusive behaviour which relates to the well‐established double‐layer concept (i.e., interfacial charge distribution) and diffusion of electroactive species. Subsequently, there are no discernible Nyquist semicircles (Figure 5b). Here, a wider low frequency region (10^−1^ to 10^2^) is likely to be subject to a greater influence of adsorption processes, associated with the adhesion of the pioneering bacteria on a conditioning film (Muñoz‐Berbel, García‐Aljaro, et al. 2008; Muñoz‐Berbel, Vigués, et al. 2008) and biofilm formation.

The EIS spectra were fitted using an equivalent circuit model (ECM) shown in Figure S4. Both the abiotic and biotic data generally had a good fit, with the quantitative fitting results shown in Table S4. *R*_s_, *R*_film_ and *R*_ct_ are the solution resistance, the resistance of the biofilm or the corrosion product film, and the charge transfer resistance, respectively. The constant phase element (CPE) characterises the ‘non‐ideal’ capacitance behaviour of either the biofilm or the corrosion product film layer, and the charge transfer capacitance. In the Table S4, *Q* and *n*, are admittance and exponent parameters from the CPE. For the abiotic control, there is initially a diffusive behaviour over the first week with a diffusive/resistive behaviour over the final 3 weeks in the film layer. Whilst there was initially a resistive behaviour over the first week in the double layer with a capacitive behaviour over the final 3 weeks, which reflects the accumulation of charge. The thin layer of ions that contacts the electrode surface serves as a dielectric, insulating the surface. The exponent parameter in the double layer reflects ideal capacitance over the final 3 weeks, which is indicative of a more prominent capacitive component. *R*_film_ has a large shift on Day 14 and again on Day 28, which indicates high *R*_p_. There are no significant changes in *R*_ct_. For the biotic test reactor, there is a capacitive behaviour in both the film layer and double layer due to the presence of the biofilm. There are no significant changes in the *R*_ct_ in the double layer over time. The exponent parameter for the film layer is greater than 0.8 at most time points, which indicates non‐ideal capacitance. This is true for the first 2 weeks in the double layer. However, during the final 2 weeks of the experiment, the exponent parameter is equal to 1 for the AR coupons in the double layer, which indicates an ideal capacitive behaviour. Whilst the exponent parameter is indicative of a diffusive behaviour for the P coupons on Days 12 and 21, with a more capacitive behaviour by Day 28. This may reflect differences in biomass of the biofilm associated with biofilm attachment and growth. The ECM and EIS both have general agreement with the LPR data.

Figure S5 shows the potentiodynamic polarisation curves for UNS G10180 for the abiotic and biotic reactors in anaerobic MB media after 28 days. Table S5 shows the corrosion parameters obtained from the polarisation curves. From the Tafel slopes, there is a decrease in cathodic current (reduction) for the biotic condition. The abiotic condition has an increased cathodic current, which indicates a greater accumulation of charge. Conversely, the anodic Tafel slopes (oxidation) are greater in the biotic compared to the abiotic media. Thus, after 28 days, the biofilm hindered the iron dissolution reactions. Overall, the abiotic condition had a higher *j*_corr_ compared to the biotic condition. This is consistent with the more uniform corrosion morphology seen for the abiotic coupon surfaces, see Figure 2. Both conditions exhibited similar *E*_corr_ on Day 28. The polarisation results corroborate the LPR and EIS data, with no significant differences when comparing between the two surface roughness types within each reactor.

### 3.5 Biofilm Characterisation

**FIGURE 6:**
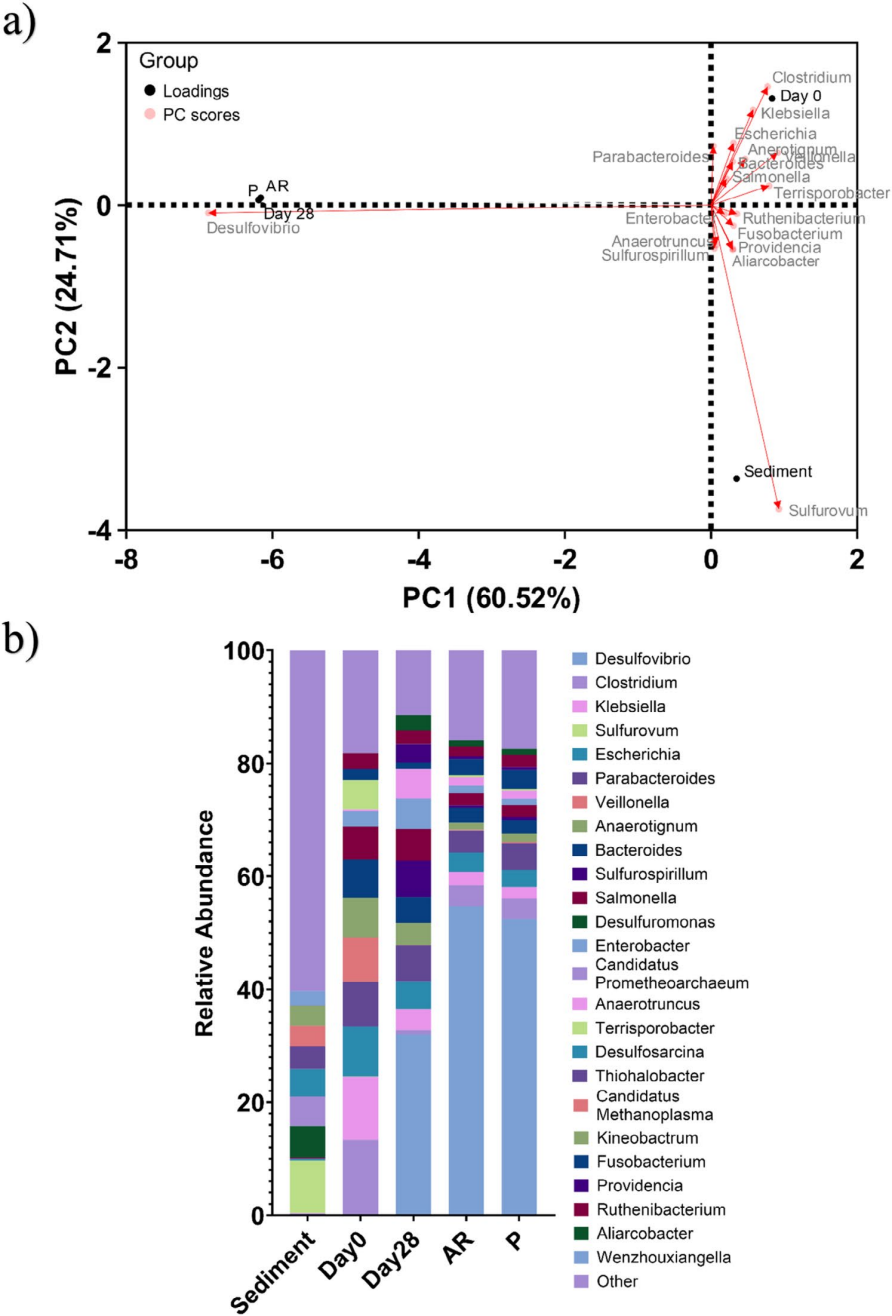
Principal component analysis biplot (a) microbial community. The results show the mean relative abundances of microbial communities classified at the genus level, for the top 25 genera, from 16S rRNA amplicon sequencing (b) for environmental marine sediment, Day 0, and Day 28 planktonic samples, AR and P biofilm samples after exposure to anaerobic MB media for 28 days.

CLSM with differentiation of live and dead biofilm cells was performed and can be found in Figure S6. The heterogeneous biofilm distribution over the surface of the CS coupons did not allow measurements of the maximum biofilm thickness. Therefore, the biofilm thickness was not determined. It was also difficult to identify significant differences in the structure and distribution of live and dead cells in the biofilms across the two surface roughness types, AR and P coupons, within the biotic reactor. In general, both surfaces had similar live and dead cell ratios (approximately 91% live to 9% dead). Active microorganism evaluation of the environmental marine sediment, the initial and final biotic MB media planktonic samples (Day 0 and Day 28), and the biotic AR and P biofilms was undertaken via 16S rRNA amplicon sequencing with two target regions, V3‐4 for Bacteria and Archaea. A total of 2,398,327 high‐quality sequences were obtained after bioinformatics processing of the raw reads. From these, 95.9% were classified for the sediment sample with 99.99% classified for the Day 0 and Day 28 planktonic samples, AR and P biofilm samples. These sequences were taxonomically classified into microbial genera. The top 25 microbial genera are presented in Table S6. Figure 6 summarises the sequencing data, showing a PCA (a) and a stacked bar plot (b) illustrating the relative abundances for the top 25 genera. Molecular identification of the microorganisms showed that the initial sediment sample had a very diverse microbial composition. Most genera had low relative abundances less than 2%. The dominant genera included *Sulfurovum*, *Desulfuromonas*, *Candidatus Prometheoarchaeum*, *Desulfosarcina*, *Thiohalobacter*, and *Candidatus Methanoplasma*, which made up approximately 33% of the total relative abundance. Interestingly, there were relatively high numbers of Archaea in the sediment sample compared to the other samples. The sediment sample had low or negative Spearman correlation coefficients (Figure S7) with the other samples. This was attributed to changes in conditions such as temperature, but primarily media composition, which was selective for the enumeration of SRB. There was much less diversity in the Day 0 sample, with *Sulfurovum*, *Candidatus Prometheoarchaeum*, *Candidatus Methanoplasma*, and *Thiohalobacter* all exhibiting negligible relative abundances, whilst genera from *Clostridium*, *Klebsiella*, *Escherichia*, *Paracabacteroides*, *Veillonella*, *Anaerotignum*, and *Terrisporobacter* made up approximately 60% of the relative abundance. The relative abundances of *Veillonella* and *Terrisporobacter* were particularly high in the initial Day 0 planktonic sample but had negligible concentrations for the Day 28 planktonic sample and both AR and P biofilms. The Day 0 planktonic had negative Spearman correlation coefficients with the sediment and Day 28 planktonic samples but had approximately 50% Spearman correlation coefficients with both AR and P biofilms. After 28 days, there was a significant shift in the microbial composition, with substantially lower abundances of methanogenic species. *Desulfovibrio* was the dominant genus, with a relative abundance of approximately 32%. *Parabacteroides*, *Sulfurospirillum*, *Salmonella*, and *Enterobacter* made up approximately another 23% of the relative abundance. Again, the Day 28 planktonic had low or negative Spearman correlation coefficients with the sediment and Day 0 planktonic samples but had approximately 50% Spearman correlation coefficients with both AR and P biofilms. The Day 28 planktonic sample had a Spearman correlation coefficient of 0.17 with the sediment sample, −0.10 with the Day 0 planktonic sample, and 0.45 with both biofilm samples. Both biofilm samples exhibited similar microbial populations on both the AR and P coupons. The relative abundances of *Desulfovibrio* increased further to approximately 55% and 53% for both AR and P biofilms respectively. *Sulfurovum*, *Candidatus Prometheoarchaeum*, *Candidatus Methanoplasma*, and *Thiohalobacter*, which were the dominant genera from the sediment sample, all had negligible relative abundances in the biofilm samples and Day 28 planktonic sample. Both biofilm samples were similar, with a Spearman correlation coefficient of 0.99. Other genera with relatively high abundances included *Clostridium*, *Klebsiella*, *Escherichia*, and *Paracabacteroides*, making up approximately 13% of the relative abundance. There were no methanogenic archaea in the biofilm samples.

The microbial activity was determined by the ATP concentrations (dissolved and total, dATP and tATP, respectively) in both the bulk fluid and biofilms, see Figure S8. For the biotic MB media (bulk fluid), there was a significant change (*p* < 0.05) in the dATP concentration when comparing Day 0 and Day 28, with dATP values increasing on the order of 10 pg mL^−1^. Also, for the biotic condition, there was a significant change (*p* < 0.05) in the concentration of tATP over the surface of the CS coupons (tATP of about 100 pg mL^−1^), when comparing between the two surface roughness types within each reactor.

## 4 Discussion

**FIGURE 7:**
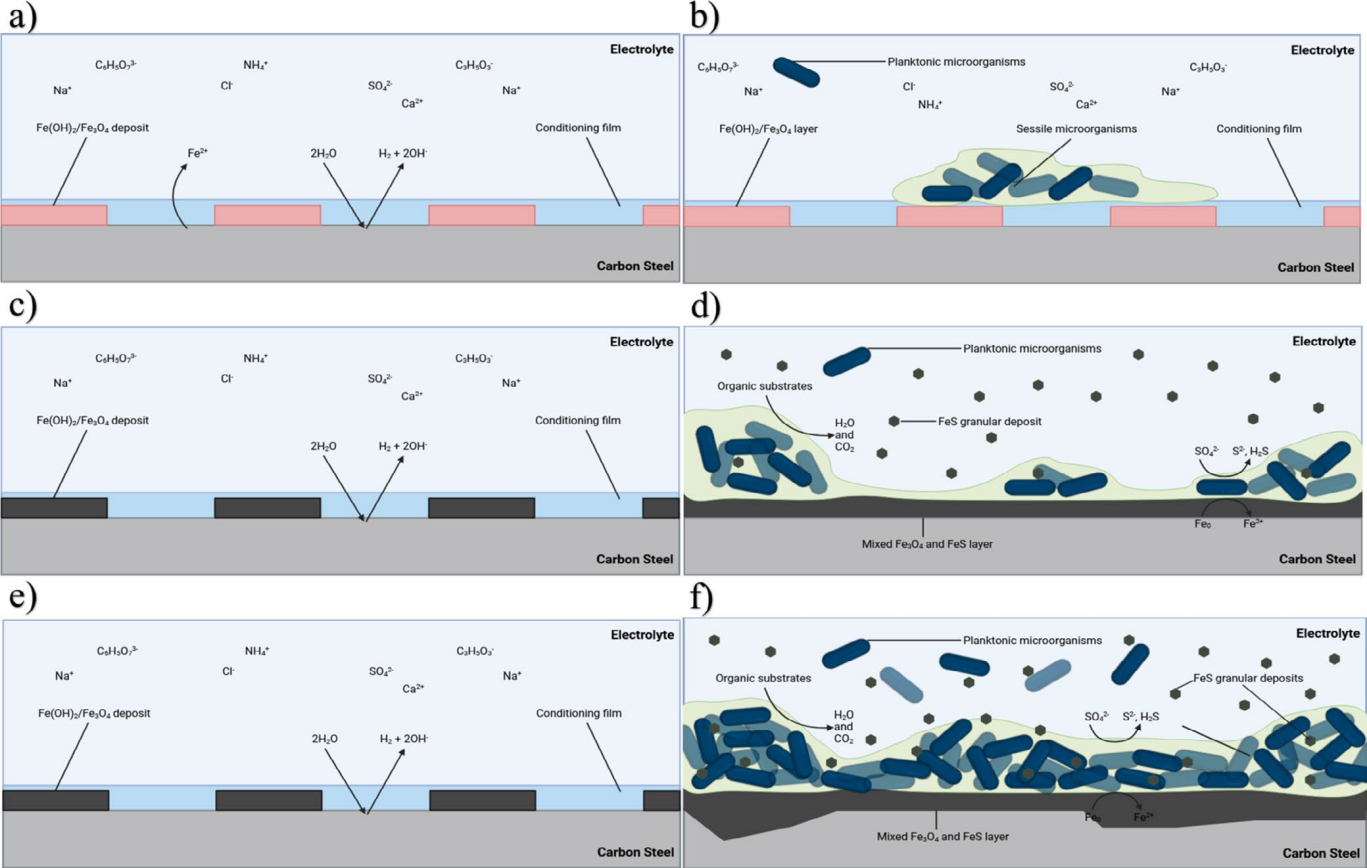
Illustration of the initial stages for UNS G10180 carbon steel in anaerobic abiotic ATCC 1249 MB media. Corrosion mechanisms, (a, b) the formation of nascent inorganic corrosion film and the organic conditioning film with pioneering bacterial attachment; (c, d) general absence of a maturing corrosion film under the abiotic condition with a prolonged period of charge accumulation; whilst there was sustained biofilm growth and colonisation for the biotic condition (e, f) moderate uniform and pitting corrosion for both conditions; under patchy corrosion deposits and a thick slimy biofilm with black granular deposits for the biotic condition. BioRender.com (2024).

Figure 7 provides an illustration of the initial stages for UNS G10180 CS in anaerobic abiotic MB media and the proposed corrosion mechanisms during the initial stages, and as they evolved over time during this present study. It highlights that abiotic environments can still form an organic film, possibly from media components, which facilitates initial charge accumulation and affects ion diffusion, despite lacking microbial activity or significant inorganic corrosion products. For this study, the abiotic surfaces were observed to have no apparent corrosion film upon retrieval; only minimal loose deposits were present. The illustration shows that in the absence of biofilm or significant corrosion products, charge accumulation persists without a protective corrosion layer forming over time. Moreover, there was no apparent turbidity within the abiotic reactor itself. As stated earlier, the abiotic condition exhibited a typical electrochemical response for the formation of a porous interface, with diffusion of soluble electroactive species across an organic conditioning film (Flemming et al. 2009). Previous works (Davydov et al. 1998; Steudel 1996; Krouse et al. 1988) often assume abiotic corrosion leads to immediate film formation (e.g., iron oxides/sulphides), but this study provides evidence that under specific anaerobic conditions, corrosion film development may be minimal or absent, with corrosion primarily governed by the HER. Due to minor oxygen ingress and in the presence of elemental sulphur, hydrogen sulphide oxidation can occur (Jones et al. 2024). Figure 7 also illustrates the proposed biotic reaction mechanisms during the initial stages of biofilm formation, and as they evolved over time on CS. This dual depiction of both biofilm structure and the evolution of corrosion morphology (from initial uniform corrosion to localised pitting under biofilm layers) offers a more dynamic and time‐resolved view than static endpoint analyses commonly reported in earlier research. Prior studies often separate electrochemical data from surface morphology. This schematic integrates both electrochemical and physical surface observations, providing a holistic, mechanistic narrative. This holistic approach, utilising MLOE, differentiates the study by offering mechanistic clarity on how and why biofilms and corrosion films evolve differently under biotic vs. abiotic conditions, and how these processes manifest in measurable electrochemical behaviour.

*Abiotic reactor*: Over the first 3 days, the abiotic condition exhibited anodic polarisation until reaching a pseudo‐steady state. The MB media contains many organic substrates, which will have adsorbed onto the electrode surface forming a conditioning film. From the ECMs, there is initially a diffusive behaviour over the 1 week. Moreover, there was initially a resistive behaviour over the first week in the double layer. The thin layer of ions that contacts the electrode surface serves as a dielectric, insulating the surface. Subsequently, there was a diffusive/resistive behaviour over the final 3 weeks in the film layer. The abiotic condition exhibits a typical electrochemical response for the formation of a porous interface, with diffusion of soluble electroactive species across an organic conditioning film (Flemming et al. 2009). Additionally, the exponent parameter in the double layer reflects ideal capacitance over the final 3 weeks, which is indicative of a more prominent capacitive component. The abiotic condition had an increased cathodic current which indicates a greater accumulation of charge. Generally, there was a moderate level of uniform corrosion of the steel surface for the abiotic media, which was consistent with the electrochemistry data. Interestingly, the abiotic surfaces had no apparent corrosion products upon retrieval. For the sterile abiotic condition, there was also generally a low $H_{2}S$ concentration.

*Biotic reactor*: Under biotic conditions, there was a gradual electronegative shift in the *E*_corr_ after the first day, until Day 9. This can be attributed to the initial attachment of microorganisms and subsequent biofilm formation on the steel surface (Tran et al. 2021). A small decrease in *R*_p_ was observed after the initial 3 day batch phase was ended, and the flow of fresh media began. However, there was generally a gradual increase in *R*_p_ over the 28 days. This was attributed to increasing sessile cell density within the biofilm. For the biotic reactor, there was a consistently uniform EIS response over the 28 day test. This suggests that there was an absence of significant detectable electrochemical changes with time. From the ECMs, there was a capacitive behaviour in both the film layer and double layer due to the presence of the biofilm. A more capacitive/diffusive behaviour was initially observed on Day 1. This is associated with the adhesion of the pioneering bacteria on a conditioning film and biofilm formation (Muñoz‐Berbel, García‐Aljaro, et al. 2008; Muñoz‐Berbel, Vigués, et al. 2008). Yet, over time there were no significant changes in the double layer. The film layer was behaving as a non‐ideal capacitor at most time points. This was also true for the first 2 weeks in the double layer. However, during the final 2 weeks of the experiment, there was some ideal capacitance behaviour specifically for the AR coupons. Conversely, there was a more diffusive behaviour for the P coupons after 2 to 3 weeks, with the capacitive behaviour only observed after 4 weeks. This may reflect differences in biomass of the biofilm associated with biofilm attachment and growth. Upon dismantling of the reactors on day 28, the biotic coupon surfaces were covered with a thick slimy biofilm but lacked the presence of a protective corrosion film. Instead, there were fine granular deposits with a dark green/black appearance, which is associated more with CMIC than EMIC.

Surface profilometry analysis further highlighted the impact of the biofilm on the CS surfaces. Generally, the shape and pit morphology were different in coupons exposed to biotic conditions compared to abiotic conditions. This difference was more pronounced when comparing the P coupons exposed to the biotic condition. Further, in this study there was a significant difference when comparing between the two surface roughness types under the biotic condition. Again, the P coupons exhibited a significantly greater *PD* compared to the AR coupons. The interactions between a surface and a bacterial cell have been shown to follow the principles of colloidal physics described by the DLVO theory (Wu et al. 2018). Depending on the topographical features and nanostructure of the material surface, the actual contact area of the material surface with the bacterial cell can either be increased or decreased (Wu et al. 2018). It is hypothesised that the AR coupons would have a greater surface area than the P coupons, and thus allow for greater surface attachment of sessile cells. Subsequently, this would translate to an increased incidence of pitting. However, in this present study the opposite was true. A more in depth analysis of the nanostructure of the coupon surfaces is therefore necessary to get a better understanding. Initially, it was hypothesised that the deterioration observed in this present study is primarily a result of CMIC, rather than EMIC due to the activity of the biofilm and the availability of lactate. CMIC of ${\mathrm{Fe}}^{0}$ by $H_{2}S$ from microbial ${\mathrm{SO}}_{4}^{2-}$ reduction has been shown to occur when organic substrates are available (Enning and Garrelfs 2014).

Analysis of the community dynamics revealed a marked change in the predominant relative abundances of microorganisms. The dominant genera from the sediment sample were generally anaerobic, halophilic, and obligately chemolithoautotrophic, obtaining energy by oxidising inorganic compounds. However, the dominant genera from the planktonic samples taken from the bulk fluid were generally facultative anaerobes known to be chemoheterotrophic, utilising organic compounds, such as lactate, through fermentation. The relative abundances of *Veillonella* and *Terrisporobacter* were particularly high in the initial Day 0 planktonic sample but had negligible concentrations after 28 Days. *Veillonella* spp., are known to require lactate for growth as they are unable to metabolise normal dietary carbohydrates (Ng and Hamilton 1971). Another microorganism of interest from the Day 0 planktonic sample was *Clostridium*. *Clostridium* spp., have genes for EET and/or demonstrated EET capabilities (Light et al. 2018; Tahernia et al. 2020). Isolates are typically fermentative electrotrophs that produce organic acids from fermentable substrates (Lovely and Holmes 2022). The MB media, for the enumeration of SRB, clearly had a large impact on the community dynamics. After 28 Days, there were significantly lower relative abundances of methanogenic species. This can be attributed to the MB media, selective for the enumeration of SRB species. Methanogens and SRP are known to compete for energy sources, with SRP outcompeting methanogens in sulphate‐rich environments (Okoro et al. 2018). Predictably, SRB were the dominant genera in both the day 28 bulk fluid and biofilm samples. *Desulfovibrio* was the dominant genera, and both biofilm samples exhibited similarly high relative abundances. Generally, the microbial populations on both the AR and P coupons were very similar. *Desulfovibrio* is a recognised electroactive corrosive microorganism, found in electromicrobiomes on the corroding surface of ${\mathrm{Fe}}^{0}$‐containing metals under anaerobic conditions. *Desulfovibrio* can extract electrons from the metallic ${\mathrm{Fe}}^{0}$ in structural materials to support anaerobic respiration (Lovely and Holmes 2022). ${\mathrm{Fe}}^{0}$ oxidation may be coupled to the reduction of electron acceptors that commonly support anaerobic respiration (Lekbach et al. 2021). Electroactivity within mixed‐species biofilm communities is an important process. Electroactivity, between fermentative electroactive bacteria (FEB) and respiratory electroactive bacteria (REB), enables food chains to be established. These electroactive microorganisms cooperate to oxidise organic compounds to ${\mathrm{CO}}_{2}$ with the reduction of ${\mathrm{Fe}}^{3+}$ (Lovely and Holmes 2022). However, this proposed direct ${\mathrm{Fe}}^{0}$‐ to‐ microorganism electron transfer has not been rigorously demonstrated in the literature, with limited examples (Tang et al. 2019, 2021). Understanding the role of EET in corrosion is critical.

**FIGURE 8:**
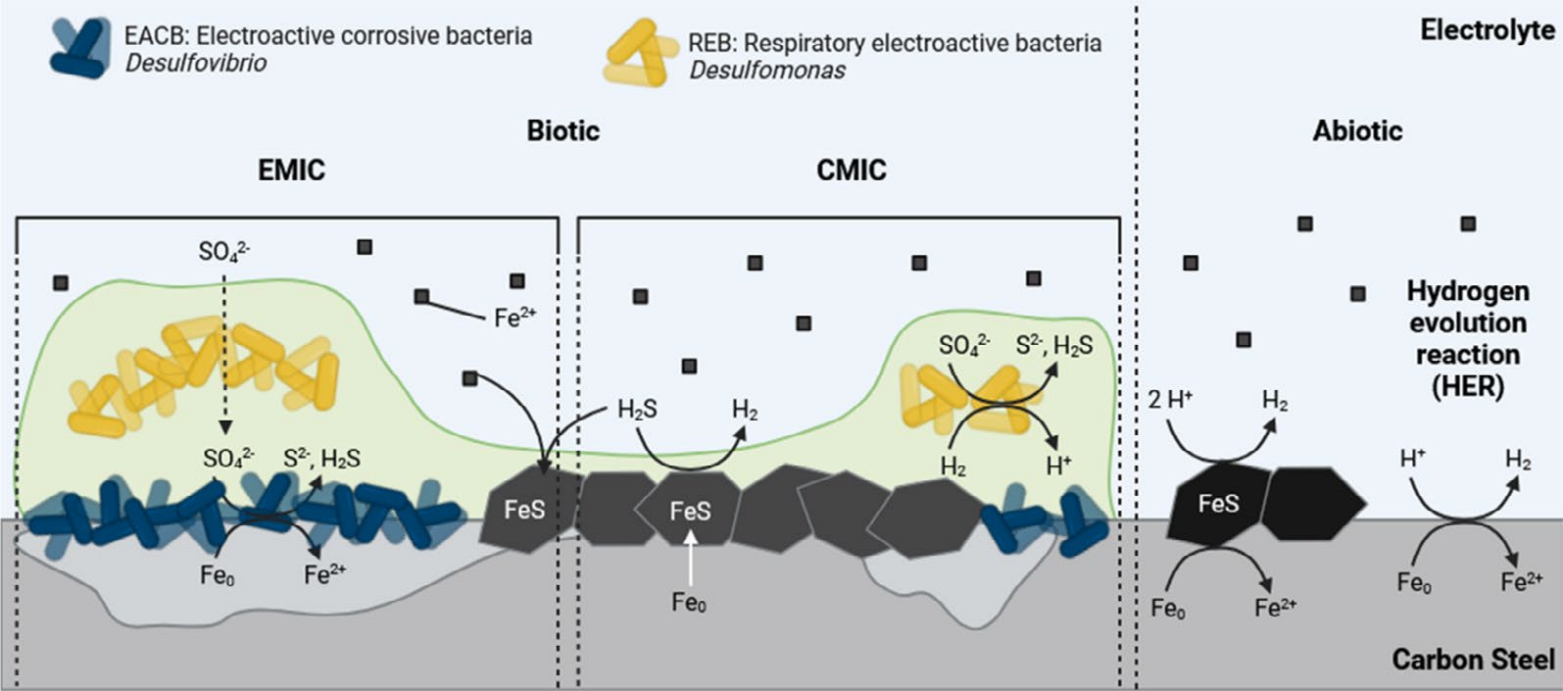
Overview of key reactions for both abiotic and biotic conditions involved in metal corrosion. Abiotic reactions were associated with the hydrogen evolution reaction, as there was a general lack of a corrosion film. Biotic reactions were associated with both EMIC and CMIC. BioRender.com (2024).

Naturally, for the biotic condition, with MB media for the enumeration of SRB, the prevailing corrosion mechanisms were both EMIC and CMIC. Figure 8 illustrates a detailed, time‐resolved overview of key corrosion reactions under abiotic and biotic conditions. Initially, CMIC may have been the dominant corrosion mechanism when $H_{2}S$ concentrations were high and organic substrates were more readily available (Jia et al. 2018). Over the first week, there was a relatively high $H_{2}S$ concentration, which gradually decreased to similar levels observed in the abiotic reactor after 2 to 3 weeks. During this time visible black particulates could be seen settling at the bottom of the reactor. After 2 weeks, there was a visible crust on the surface of the MB media. Moreover, the media was black in appearance with increased levels of turbidity. EMIC, via the direct uptake of electrons from the metal surface (Enning and Garrelfs 2014), may have been the primary corrosion mechanism during this period. The increased turbidity was attributed to a combination of increased ${\mathrm{Fe}}^{2+}$ ions and fine suspensions of FeS produced in the bulk fluid rather than as a film on metal surface. This was due to the presence of a thick biofilm which covered a greater surface area than corrosion products on the metal surface. Additionally, the decreasing concentration of $H_{2}S$ may also be attributed to the large headspace within the reactor system. A large headspace has been shown in other studies to allow for more $H_{2}S$ to escape from the bulk fluid. In turn, the environment within the bulk fluid becomes less cytotoxic further supporting biofilm growth (Jia et al. 2018). This could have also contributed to a lack of FeS passivation on the metal surface. Conversely, the concentration of $H_{2}S$ is likely higher under the biofilm compared to the bulk fluid due to cell density. Furthermore, the biofilm is a diffusion barrier that slows down the escape of $H_{2}S$. Thus, CMIC could still be a contributing factor to localised pitting corrosion. However, organic substrates are likely at lower concentrations under the biofilm. Based on visual observation of the coupons taken from the biotic condition, there was an inorganic corrosion product on the metal surface, but there were also distinct dark green/black granular deposits present. EMIC by SRB is characterised by the formation of large amounts of inorganic corrosion products (Enning and Garrelfs 2014). However, the biotic surfaces were covered with a thick slimy biofilm. While prior research often focuses on one dominant MIC mechanism (either CMIC or EMIC) (Enning and Garrelfs 2014; Jia et al. 2018), this study is novel in mapping the dynamic interplay and temporal succession of CMIC to EMIC, supported by real‐time observations. Moreover, prior MIC studies overlook gas‐phase interactions or reactor design influence, whereas this study highlights how reactor configuration affects microbial activity, chemical gradients, and the balance between CMIC and EMIC. Previous studies often focus on FeS layer formation as a passivating factor in MIC, but this study proposes that biofilm architecture itself prevents FeS passivation, maintaining active corrosion sites. This dual interaction of biofilm structure and corrosion product dynamics is underexplored in prior literature. This nuanced focus on biofilm‐controlled microenvironments versus bulk conditions (and how they diverge over time) is a novel aspect, contrasting with many studies that treat bulk fluid measurements as representative of conditions at the metal surface. Figure 8 clarifies how dynamic environmental shifts and biofilm behaviour interplay to control the balance between CMIC and EMIC, offering new insights into localised pitting progression.

In sulphate‐containing anoxic environments, previous studies have demonstrated that CMIC and EMIC are the likely primary processes that drive ${\mathrm{Fe}}^{0}$ corrosion (Enning and Garrelfs 2014). However, determining the relative contributions of EMIC and CMIC in different anoxic environments is challenging. Moreover, there are certain situations in which SRB‐induced biocorrosion can be further exacerbated (Enning and Garrelfs 2014). For example, ingress of molecular $O_{2}$ (Hardy and Brown 1984; Jack et al. 1998; Lee et al. 1993) into previously anoxic systems can lead to the formation of highly corrosive sulphur species from the partial oxidation of dissolved $H_{2}S$ and biogenic FeS deposits on steel surfaces (MacDonald et al. 1978; Nielsen et al. 1993). CMIC and EMIC produce corrosion products with inherently different relative amounts of sulphidic (containing $H_{2}S$, ${\mathrm{HS}}^{-}$, $S^{2-}$) and non‐sulphidic iron. FeS are the characteristic products of SRB‐induced corrosion (Enning and Garrelfs 2014). While CMIC produces FeS as the sole mineral product, FeS has been found to account for only about 25% of the total iron minerals formed by the EMIC mechanism (Jia et al. 2018, 2017). Biogenic dissolved $H_{2}S$ have been shown to be a key determining factor in whether CMIC or EMIC are the primary corrosion mechanism (Jia et al. 2018). Jia et al. showed that a larger headspace volume resulted in lower dissolved $H_{2}S$, thereby reducing cytotoxicity within the bulk fluid. This allowed for greater SRB growth resulting in increased sessile cell densities and more severe MIC. It was hypothesised that direct electron uptake via EMIC rather than biogenic $H_{2}S$ metabolites produced via CMIC was the primary corrosion mechanism during the later stages of this study (Jia et al. 2018). There have been several studies performed with pure laboratory‐grown SRB cultures that have reported lower *CR*s in media with organic electron donors (Booth 1964; Cord‐Ruwisch 2000; Beech et al. 1994; Gaylarde 1992; Nielsen et al. 1993). Similarly, these studies have speculated that the presence of microorganisms capable of EMIC play a critical role in MIC. EMIC is hypothesised to be a widespread mechanism among corrosive microorganisms (Enning et al. 2012; Dinh et al. 2004).

The presence of biogenic FeS appears to also play a central role in CMIC and EMIC. Newman et al. stated that formation of a protective FeS film usually occurs at high concentrations of dissolved $H_{2}S$ which exceed the concentration of dissolved ${\mathrm{Fe}}^{2+}$ ions at the unreacted metal surface (Newman et al. 1992). The presence of biogenic FeS may hinder further ${\mathrm{Fe}}^{0}$ corrosion. The formation of a tightly adherent FeS film on the metal surface may act as a passive layer (Newman et al. 1992; Sun and Nešic 2007). Such films act as an effective process barrier by impeding the diffusion of ${\mathrm{Fe}}^{2+}$ ions from the metal anode to the aqueous environment (Enning and Garrelfs 2014; Newman et al. 1992). Several studies have observed impediment of the anodic half‐reaction in cultures of SRB grown in the presence of organic substrates (Booth and Tiller 1960; Tiller and Booth 1962; Tiller 1983). However, in organic matter‐free cultures, where the predominant corrosive mechanism is EMIC, no significant slowdown of corrosion due to crust formation has been observed to date (Enning and Garrelfs 2014). Enning et al. demonstrated that the bulky black crusts formed through EMIC are electrically conductive (Enning et al. 2012). Electrons can flow from the corroding ${\mathrm{Fe}}^{0}$ through the electroconductive mineral crust to the crust‐attached cells reducing ${\mathrm{SO}}_{4}^{2-}$ (Enning and Garrelfs 2014; Enning et al. 2012). Yet, disruption of the FeS film can occur. CMIC has been demonstrated in lactate‐based media with high concentrations of ${\mathrm{Fe}}^{2+}$ salts, to prevent the formation of the protective FeS film and instead deposit fine suspensions of the mineral on the metal (Enning and Garrelfs 2014; Lee and Characklis 1993). Rupture of the FeS film and local re‐exposure of metallic ${\mathrm{Fe}}^{0}$ subsequently results in rapid pitting corrosion. Understanding the relative contributions of EMIC and CMIC within mixed‐species biofilms is critical, as given the dual role of FeS films in corrosion, resulting *CR* in sulphidic SRB cultures can vary significantly.

Unlike many prior MIC investigations (Enning and Garrelfs 2014; Jia et al. 2018; Enning et al. 2012), this study uses a marine sediment‐derived microbial consortium, closely mimicking natural, ecologically relevant conditions. The sediment community initially contained diverse taxa such as *Sulfurovum*, *Desulfuromonas*, and methanogenic archaea. However, selective pressures applied by the MB media, reactor conditions, and available substrates drove a succession towards SRB dominance, particularly *Desulfovibrio* spp., which became the predominant genus in both the biofilm and planktonic phases after 28 days. Early stages showed enrichment of fermentative anaerobes. However, over‐time sulphate‐reducing, electroactive taxa dominated. Many prior studies do not capture or report such dynamic ecological succession over time. Methanogenic species, initially abundant in sediment, were almost entirely absent in the mature biofilms, consistent with SRB outcompeting methanogens in sulphate‐rich environments. This ecological dynamic is well‐documented theoretically (An et al. 2021; Huang et al. 2024) but is empirically demonstrated here. We also clearly delineate the mechanistic transition between CMIC and EMIC here. This study provides a time‐resolved view of how mixed microbial communities adapt under continuous flow, anaerobic conditions, and selective pressures. The dual biofilm reactor system allowed clear observation of a dynamic interplay between CMIC (early stage, high $H_{2}S$, abundant organics) and EMIC (later stages, thick biofilm, direct electron uptake by *Desulfovibrio* spp.), with temporal shifts linked to microbial community changes and environmental factors like headspace volume. Many studies isolate EMIC or CMIC mechanisms (Enning and Garrelfs 2014; Jia et al. 2018), often overlooking their potential co‐occurrence or evolution over time within natural, mixed‐species biofilms. Here, we highlight how community composition shifts correlate to changes in prevailing corrosion mechanisms, particularly under continuous, realistic conditions. CMIC predominates in the early stages, driven by high concentrations of biogenic $H_{2}S$ and abundant organic substrates. EMIC becomes more prominent in later stages, as sulphate‐reducing, electroactive Desulfovibrio spp. dominate the biofilm. This coincides with increased pit density and the formation of non‐passivating, fine corrosion products, distinct from classical FeS crusts. Additionally, we propose that biofilm architecture may play an underappreciated role in suppressing FeS passivation, maintaining exposed reactive metal surfaces and prolonging EMIC processes. This challenges classical models that assume biogenic sulphide passivates corrosion via FeS crust formation, offering a new conceptual framework for localised MIC control strategies. Contrary to expectations and many prior reports, the P coupons exhibited significantly higher pit density and more severe localised corrosion than AR coupons, despite theoretically offering lower surface area for microbial attachment. This suggests that under mixed‐species biofilm conditions, biofilm attachment and corrosion may not strictly follow simplified models of surface roughness effects, possibly influenced by biofilm biomass distribution, microbial motility, and localised chemistry, an area warranting further investigation.

## 5 Conclusions

This study presents a novel and ecologically relevant platform for investigating MIC mechanisms under controlled but realistic conditions. By integrating MLOE, we delineate the temporal evolution of corrosion processes from CMIC to EMIC within a mixed‐species SRB‐dominated biofilm. The approach allows for mechanistic resolution that is often obscured in more reductionist systems and highlights the importance of microbial succession, chemical gradients, and biofilm structure in influencing corrosion outcomes. This customizable dual bioreactor system enables the realistic simulation of industrial conditions, such as modulating flow rates to replicate stagnant versus high‐shear environments, adjusting redox potential via controlled gas sparging (e.g., nitrogen, hydrogen, or low‐level oxygen), and varying substrate types (e.g., lactate, acetate, ethanol) to mimic different organic load scenarios, thereby providing a robust platform for evaluating MIC mitigation strategies, including the performance and persistence of biocides under dynamic, ecologically relevant conditions (Jones et al. 2025).

- The environmental conditions in this study supported continuous biofilm growth. Electrochemical methods were concurrently employed to identify the onset of biofilm attachment and formation, as well as to assess the biofilm's impact on the surface of the CS coupons.
- Surface profilometry in this study revealed that biotic conditions resulted in a significantly higher *PD* (*p* < 0.05 for abiotic and biotic AR coupons, *p* < 0.0001 for abiotic and biotic P coupons, *p* < 0.0066 for biotic AR and biotic P coupons) with deeper and larger pits compared to sterile abiotic conditions.
- Additionally, biofilm characterisation through sequencing indicated a significant increase in SRB, particularly the electroactive and corrosive *Desulfovibrio* spp., within the biofilm. This microorganism plays a crucial role in EET, a key process in MIC.

These findings contribute to a deeper understanding of the relative contributions of EMIC and CMIC within a mixed‐species biofilm. Identifying the microbial mechanisms that drive the corrosion of CS under anoxic conditions at the metal/electrolyte interface is essential, as *CR*s in sulphidic SRB cultures can vary widely. By employing MLOE, a comprehensive understanding of the role SRB play in MIC can be developed. In turn, this can inform the design of more sustainable prevention and mitigation strategies.

## Author Contributions

**Liam Jones:** conceptualisation, data curation, formal analysis, investigation, methodology, project administration, validation, visualisation, writing – original draft, writing – review and editing. **Maria Salta:** conceptualisation, writing – review and editing, supervision, project administration, funding acquisition. **Torben Lund Skovhus:** conceptualisation, writing – review and editing, supervision, project administration, funding acquisition. **Kathryn Thomas:** conceptualisation, writing – review and editing, supervision, project administration, funding acquisition. **Timothy Illson:** conceptualisation, writing – review and editing, supervision, project administration, funding acquisition. **Julian Wharton:** conceptualisation, writing – review and editing, supervision, project administration, funding acquisition. **Jeremy Webb:** conceptualisation, writing – review and editing, supervision, project administration, funding acquisition.

## Conflicts of Interest

The authors declare no conflicts of interest.

## Supporting information


**Data S1.** Supporting Information.

## Data Availability

The sequencing data generated in this study have been deposited in the NCBI Sequence Read Archive (SRA) under the accession number PRJNA1238084. Additional data supporting the findings of this study are available from the corresponding author upon reasonable request.

## References

[emi70116-bib-0001] Al‐Abbs, F. , R. Bhola , J. Spear , D. Olson , and B. Mishra . 2013. “Electrochemical Characterization of Microbiologically Influenced Corrosion on Line Pipe Steel Exposed to Faculative Anaerobic Desulfovibrio sp.” International Journal of Electrochemical Science 8: 859–871.

[emi70116-bib-0002] Al‐Abbs, F. , C. Williamson , S. Bhola , et al. 2013a. “Influence of Sulfate Reducing Bacterial Biofilm on Corrosion Behavior of Low‐Alloy, High‐ Strength Steel (API‐5L X80).” International Biodeterioration & Biodegradation 78: 34–42.

[emi70116-bib-0003] Al‐Abbs, F. , C. Williamson , S. Bhola , et al. 2013b. “Microbial Corrosion in Line Pipe Steel Under the Influence of Sulfate Reducing Consortium Isolated From an Oil Field.” Journal of Materials Engineering and Performance 22, no. 11: 3517–3529.

[emi70116-bib-0004] An, B. , E. Deland , O. Sobol , J. Yao , T. Skovhus , and A. Koerdt . 2021. “The Differences in the Corrosion Product Compositions of Methanogen‐Induced Microbiologically Influenced Corrosion (Mi‐MIC) Between Static and Dynamic Growth Conditions.” Corrosion Science 180: 109179.

[emi70116-bib-0005] ASTM G1‐03 . 2018. Standard Practice for Preparing, Cleaning, and Evaluationg Corrosion Test Specimens. ASTM International.

[emi70116-bib-0006] ASTM G48‐11 . 2020. Standard Test Methods for Pitting and Crevice Corrosion Resistance of Stainless Steels and Related Alloys by Use of Ferric Chloride Solution. ASTM International.

[emi70116-bib-0007] Beech, I. , C. Cheung , C. Chan , M. Hill , R. Franco , and A. Lino . 1994. “Study of Parameters Implicated in the Biodeterioration of Mild Steel in the Presence of Different Species of Sulfate‐Reducing Bacteria.” International Biodeterioration & Biodegradation 34: 289–303.

[emi70116-bib-0008] Booth, G. 1964. “Sulphur Bacteria in Relation to Corrosion.” Journal of Applied Bacteriology 27: 174–181.

[emi70116-bib-0009] Booth, G. , and A. Tiller . 1960. “Polarization Studies of Mild Steel in Cultures of Sulphate‐Reducing Bacteria.” Transactions of the Faraday Society 56: 1689–1696.

[emi70116-bib-0010] Booth, G. , and A. Tiller . 1962. “Polarization Studies of Mild Steel in Cultures of Sulphate‐Reducing Bacteria. Part 3: Halophilic Organisms.” Transactions of the Faraday Society 58: 2510–2516.

[emi70116-bib-0011] Cord‐Ruwisch, R. 2000. “Microbially Influenced Corrosion of Steel.” In Environmental Microbe‐Metal Interactions, 159–173. ASM Press.

[emi70116-bib-0012] Costello, J. 1974. “Cathodic Depolarization by Sulphate‐Reducing Bacteria.” South African Journal of Science 70: 202–204.

[emi70116-bib-0013] Davydov, A. , K. Chuang , and A. Sanger . 1998. “Mechanism of H2S Oxidation by Ferric Oxide and Hydroxide Surfaces.” Journal of Physical Chemistry B 102: 4745–4752.

[emi70116-bib-0014] Dinh, H. , J. Kuever , M. Mußmann , A. Hassel , M. Stratmann , and F. Widdel . 2004. “Iron Corrosion by Novel Anaerobic Microorganisms.” Nature 427: 829–832. 14985759 10.1038/nature02321

[emi70116-bib-0015] Enning, D. , and J. Garrelfs . 2014. “Corrosion of Iron by Sulfate‐Reducing Bacteria: New Views of an Old Problem.” Applied and Environmental Microbiology 80: 1226–1236. 24317078 10.1128/AEM.02848-13 PMC3911074

[emi70116-bib-0016] Enning, D. , H. Venzlaff , J. Garrelfs , et al. 2012. “Marine Sulfate‐Reducing Bacteria Cause Serious Corrosion of Iron Under Electroconductive Biogenic Mineral Crust.” Environmental Microbiology 14, no. 7: 1772–1787. 22616633 10.1111/j.1462-2920.2012.02778.x PMC3429863

[emi70116-bib-0017] Flemming, H.‐C. , P. S. Murthy , R. Venkatesan , and K. Cooksey . 2009. Marine and Industrial Biofouling. Springer. 978‐3‐540‐69794‐7.

[emi70116-bib-0018] Gaylarde, C. 1992. “Sulfate‐Reducing Bacteria Which Do Not Induce Accelerated Corrosion.” International Biodeterioration & Biodegradation 30: 331–338.

[emi70116-bib-0019] Hamilton, W. A. 1985. “Sulphate‐Reducing Bacteria and Anaerobic Corrosion.” Annual Review of Microbiology 39: 195–217. 10.1146/annurev.mi.39.100185.001211 3904600

[emi70116-bib-0020] Hardy, J. 1983. “Utilisation of Cathodic Hydrogen by Sulphate‐Reducing Bacteria.” British Corrosion Journal 18: 190–193.

[emi70116-bib-0021] Hardy, J. , and J. Brown . 1984. “The Corrosion of Mild Steel by Biogenic Sulfide Films Exposed to Air.” Corrosion 40: 650–654.

[emi70116-bib-0022] Huang, Q. , Y. Liu , and B. Dhar . 2024. “Deciphering the Microbial Interactions and Metabolic Shifts at Different COD/Sulfate Ratios in Electro‐Assisted Anaerobic Digestion.” Journal of Hazardous Materials 480: 135801. 39270585 10.1016/j.jhazmat.2024.135801

[emi70116-bib-0023] Jack, T. , A. Wilmott , J. Stockdale , G. Van Bouven , R. Worthingham , and R. Sutherby . 1998. “Corrosion Consequences of Secondary Oxidation of Microbial Corrosion.” Corrosion 54: 246–252.

[emi70116-bib-0024] Jia, R. , Y. Li , H. Al‐Mahamedh , and T. Gu . 2017. “Enhanced Biocide Treatments With D‐Amino Acid Mixtures Against a Biofilm Consortium From a Water Cooling Tower.” Frontiers in Microbiology 8: 1538. 28861053 10.3389/fmicb.2017.01538 PMC5561659

[emi70116-bib-0025] Jia, R. , J. Tan , P. Jin , D. Blackwood , D. Xu , and T. Gu . 2018. “Effects of Biogenic H2S on the Microbiologically Influenced Corrosion of C1018 Carbon Steel by Sulfate Reducing *Desulfovibrio Vulgaris* Biofilm.” Corrosion Science 130: 1–11.

[emi70116-bib-0026] Jones, L. , M. Salta , T. Lund Skovhus , et al. 2025. “Toward Simulating Offshore Oilfield Conditions: Insights Into Microbiologically Influenced Corrosion From a Dual Anaerobic Biofilm Reactor.” Applied and Environmental Microbiology 91, no. 6: e02221–e02224. 40035601 10.1128/aem.02221-24 PMC12175496

[emi70116-bib-0027] Jones, L. , M. Salta , T. Skovhus , et al. 2024. “Dual Anaerobic Reactor Model to Study Biofilm and Microbiologically Influenced Corrosion Interactions on Carbon Steel.” Npj Materials Degradation 8: 125. 39649128 10.1038/s41529-024-00542-x PMC11621017

[emi70116-bib-0028] Knisz, J. , R. Eckert , L. M. Gieg , et al. 2023. “Microbiologically Influenced Corrosion—More Than Just Microorganisms.” FEMS Microbiology Reviews 47, no. 5: 1–33. 10.1093/femsre/fuad041 PMC10479746 37437902

[emi70116-bib-0029] Krouse, H. , C. Viau , L. Eliuk , A. Ueda , and S. Halas . 1988. “Chemical and Isotopic Evidence of Thermochemical Sulphate Reduction by Light Hydrocarbon Gases in Deep Carbonate Reservoirs.” Nature 333: 415–419.

[emi70116-bib-0030] Lee, J. S. , and B. Little . 2015. “Yeast Extract, Technical Note: Electrochemical and Chemical Complications Resulting From Yeast Extract Addition to Stimulate Microbial Growth.” Corrosion 71, no. 12: 1434–1440.

[emi70116-bib-0031] Lee, W. , and W. Characklis . 1993. “Corrosion of Mild Steel Under Anaerobic Biofilm.” Corrosion 49, no. 3: 186–199.

[emi70116-bib-0032] Lee, W. , Z. Lewandowski , M. Morrison , W. G. Characklis , R. Avci , and P. H. Nielsen . 1993. “Corrosion Ofmild Steel Underneath Aerobic Biofilms Containing Sulfate‐Reducing Bacteria. Part I: At High Dissolved Oxygen Concentrations.” Biofouling 7: 217–239.

[emi70116-bib-0033] Lee, W. , Z. Lewandowski , P. Nielsen , and W. Hamilton . 1995. “Role of Sulfate‐Reducing Bacteria in Corrosion of Mild Steel: A Review.” Biofouling 8: 165–194.

[emi70116-bib-0034] Lekbach, Y. , T. Liu , Y. Li , et al. 2021. “Chapter Five—Microbial Corrosion of Metals: The Corrosion Microbiome.” In Advances in Microbial Physiology, vol. 78, 317–390. Elsevier. 34147188 10.1016/bs.ampbs.2021.01.002

[emi70116-bib-0035] Light, S. , L. Su , R. Rivera‐Lugo , et al. 2018. “A Flavin‐Based Extracellular Electron Transfer Mechanism in Diverse Gram‐Positive Bacteria.” Nature 562: 140–144. 30209391 10.1038/s41586-018-0498-z PMC6221200

[emi70116-bib-0036] Lovely, D. R. , and D. E. Holmes . 2022. “Electromicrobiology: The Ecophysiology of Phylogenetically Diverse Electroactive Microorganisms.” Nature Reviews. Microbiology 20: 5–19. 34316046 10.1038/s41579-021-00597-6

[emi70116-bib-0037] MacDonald, D. , B. Roberts , and J. Hyne . 1978. “Corrosion of Carbon Steel During Cyclical Exposure to Wet Elemental Sulfur and Atmosphere.” Corrosion Science 18: 499–501.

[emi70116-bib-0038] Muñoz‐Berbel, X. , C. García‐Aljaro , and F. Muñoz . 2008. “Impedimetric Approach for Monitoring the Formation of Biofilms on Metallic Surfaces and the Subsequent Application to the Detection of Bacteriophages.” Electrochimica Acta 53: 5739–5744.

[emi70116-bib-0039] Muñoz‐Berbel, X. , N. Vigués , A. Jenkins , J. Mas , and F. Muñoz . 2008. “Impedimetric Approach for Quantifying Low Bacteria Concentrations Based on the Changes Produced in the Electrode–Solution Interface During the Pre‐Attachment Stage.” Biosensors and Bioelectronics 23: 1540–1546. 18308537 10.1016/j.bios.2008.01.007

[emi70116-bib-0040] NACE SP0775‐2023 . 2023. Preparation, Installation, Analysis, and Interpretation of Corrosion Coupons in Hydrocarbon Operations. AMPP, NACE Standards.

[emi70116-bib-0042] Newman, R. , K. Rumash , and B. Webster . 1992. “The Effect of Pre‐Corrosion on the Corrosion Rate of Steel in Neutral Solutions Containing Sulfide: Relevance to Microbially Influenced Corrosion.” Corrosion Science 33: 1877–1884.

[emi70116-bib-0043] Newman, R. , B. Webster , and R. Kelly . 1991. “The Electrochemistry of SRB Corrosion and Related Inorganic Phenomena.” ISIJ International 31: 201–209.

[emi70116-bib-0044] Ng, S. , and I. Hamilton . 1971. “Lactate Metabolism by *Veillonella Parvula* .” Journal of Bacteriology 105: 999–1005. 4323300 10.1128/jb.105.3.999-1005.1971 PMC248529

[emi70116-bib-0045] Nielsen, P. , W. Lee , Z. Lewandowski , M. Morison , and W. Characklis . 1993. “Corrosion of Mild Steel in an Alternating Oxic and Anoxic Biofilm System.” Biofouling 7: 267–284.

[emi70116-bib-0046] Okoro, C. C. , E. E. Nwezza , and J. Lin . 2018. “Persistence of Halophilic Methanogens and Oil‐Degrading Bacteria in an Offshore Oil‐Producing Facility.” Geomicrobiology Journal 35: 323–333.

[emi70116-bib-0047] Rabalais, N. , R. Turner , D. Justic´ , Q. Dortch , and W. Wiseman . 1999. Characterization of Hypoxia. NOAA Coastal Ocean Program.

[emi70116-bib-0048] Schiraldi, C. , and M. De Rosa . 2014. “Mesophilic Organisms.” In Encyclopedia of Membranes, edited by E. Drioli and L. Giorno , 1–2. Springer.

[emi70116-bib-0049] Sorokin, Y. 1966. “Role of Carbon Dioxide and Acetate in Biosynthesis by Sulphate‐Reducing Bacteria.” Nature 210: 551–552. 5960530 10.1038/210551a0

[emi70116-bib-0050] Steudel, R. 1996. “Mechanism for the Formation of Elemental Sulfur From Aqueous Sulfide in Chemical and Microbiological Desulfurization Processes.” Industrial and Engineering Chemistry Research 35: 1417–1423.

[emi70116-bib-0051] Sun, W. , and S. Nešic . 2007. A Mechanistic Model of H2S Corrosion of Mild Steel, Paper 07655. NACE International.

[emi70116-bib-0052] Tahernia, M. , E. Plotkin‐Kaye , M. Mohammadifar , et al. 2020. “Characterization of Electrogenic Gut Bacteria.” ACS Omega 5, no. 45: 29439–29446. 33225175 10.1021/acsomega.0c04362 PMC7676329

[emi70116-bib-0053] Tang, H. , D. Holmes , T. Ueki , and P. Palacios . 2019. “Iron Corrosion via Direct Metal‐Microbe Electron Transfer.” MBio 10, no. 3: e00303–e00319. 31088920 10.1128/mBio.00303-19 PMC6520446

[emi70116-bib-0054] Tang, H. , C. Yang , T. Ueki , et al. 2021. “Stainless Steel Corrosion via Direct Iron‐To‐Microbe Electron Transfer by Geobacter Species.” ISME Journal 10: 3084–3093. 10.1038/s41396-021-00990-2 PMC8443633 33972726

[emi70116-bib-0055] Tiller, A. 1983. “Electrochemical Aspects of Corrosion: An Overview.” In Microbial Corrosion, 54–65. Metals Society.

[emi70116-bib-0056] Tiller, A. , and G. Booth . 1962. “Polarization Studies of Mild Steel in Cultures of Sulphate‐Reducing Bacteria. Part 2. Thermophilic Organisms.” Transactions of the Faraday Society 58: 110–115.

[emi70116-bib-0057] Tran, T. , K. Kannoorpatti , A. Padovan , S. Thennadil , and K. Nguyen . 2021. “Microbial Corrosion of DSS 2205 in an Acidic Chloride Environment Under Continuous Flow.” PLoS One 16, no. 5: e0251524. 33979409 10.1371/journal.pone.0251524 PMC8115847

[emi70116-bib-0058] Venzlaff, H. , D. Enning , J. Srinivasan , et al. 2013. “Accelerated Cathodic Reaction in Microbial Corrosion of Iron due to Direct Electron Uptake by Sulfate‐Reducing Bacteria.” Corrosion Science 66: 88–96.

[emi70116-bib-0059] Wu, S. , S. Altenreid , A. Zogg , F. Zuber , K. Maniura‐Weber , and Q. Ren . 2018. “Role of the Surface Nanoscale Roughness of Stainless Steel on Bacterial Adhesion and Microcolony Formation.” ACS Omega 3: 6456–6464. 30023948 10.1021/acsomega.8b00769 PMC6045408

[emi70116-bib-0060] Xu, D. , and T. Gu . 2014. “Carbon Source Starvation Triggered More Aggressive Corrosion Against Carbon Steel by the *Desulfovibrio vulgaris* Biofilm.” International Biodeterioration & Biodegradation 91: 74–81.

[emi70116-bib-0061] Xu, D. , Y. Li , F. Song , and T. Gu . 2013. “Laboratory Investigation of Microbiologically Influenced Corrosion of C1018 Carbon Steel by Nitrate Reducing Bacterium *Bacillus licheniformis* .” Corrosion Science 77: 385–390.

