# Supplementary material for: Effects of Sulphate‐Reducing Bacteria Mixed‐Species Biofilms on Microbiologically Influenced Corrosion
Source: Environ Microbiol. 2025 Aug 20;27(8):e70116. doi: 10.1111/1462-2920.70116 (PMC12366542; doi:10.1111/1462-2920.70116)
Supplement: Supplementary file 1 — Data S1. Supporting Information. [file EMI-27-e70116-s001.docx]

# Supplementary Material

**Supplementary Table 1.** ATCC 1249 Modified Baar’s media composition. Each component is adjusted to pH 7.5 and autoclaved at 121°C. The three components are mixed aseptically and stored under anaerobic conditions. The anaerobic chamber gas mixture consisted of 85% N_2_, 10% CO_2_ and 5% H_2_.

| **Component 1** | |
| --- | --- |
| MgSO_4_ | 2.0 g |
| Sodium Citrate | 5.0 g |
| CaSO_4_ x 2H_2_O | 1.0 g |
| NH_4_Cl | 1.0 g |
| DI Water | 400.0 ml |
| **Component 2** | |
| K_2_HPO_4_ | 0.5 g |
| DI Water | 200.0 ml |
| **Component 3** | |
| Sodium Lactate | 3.5 g |
| Yeast Extract | 1.0 g |
| DI Water | 400.0 ml |

| **(a)** **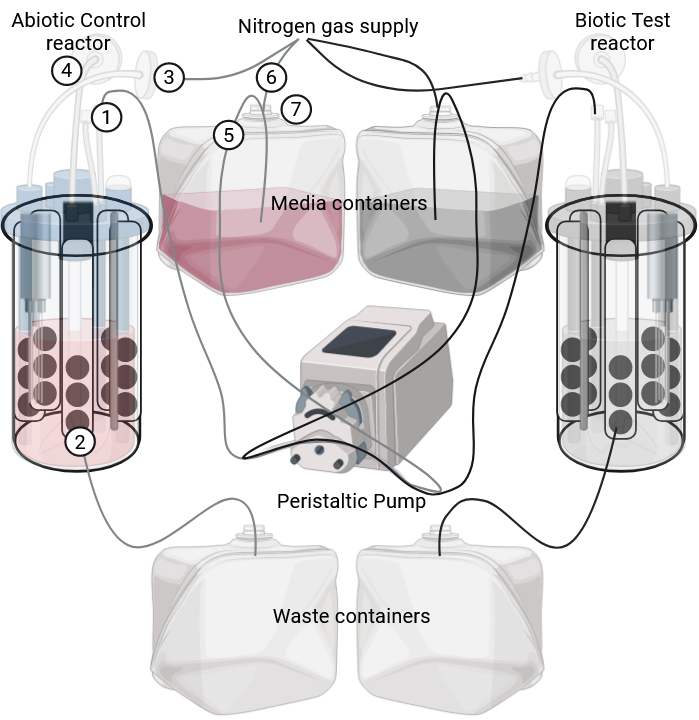** | **(b)**  **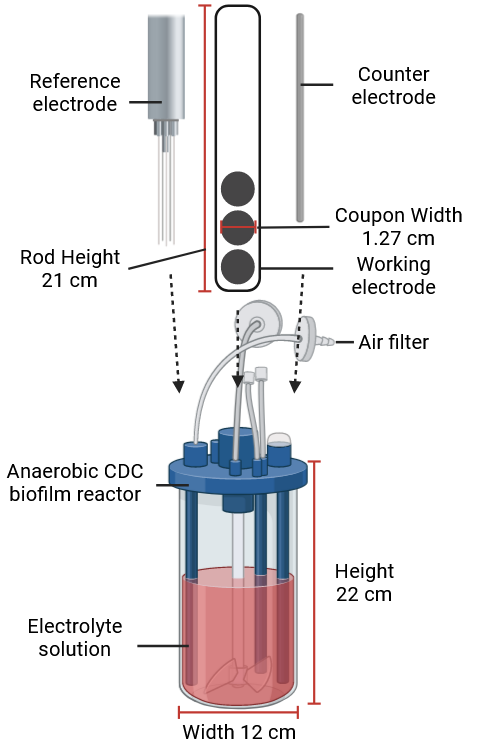** |
| --- | --- |

**Supplementary Figure 1.** **(a)** The dual anaerobic biofilm reactor system (abiotic and biotic reactors) comprising 10 L media containers, peristaltic pump, magnetic stirrer/hot plate, sulphide microsensor, and the three electrode cell setup. Each reactor has five rods, with three coupons in each rod (15 coupons in total). Each reactor has four inlets. ➀ The first inlet is connected to the peristaltic pump and then the media container. ➁ The outlet is connected to the waste containers. ➂ Connection to the nitrogen gas source. ➃ Air filter (Millex, 0.2 µm) that acts as the exit for excess gas in the reactors. ➄ The 10 L media container is connected via the peristaltic pump and feeds the first inlet in the reactor. ➅ Connection to the nitrogen gas source. ➆ Air filter (Millex, 0.2 µm) that acts as the exit for excess gas in the media containers. **(b)** detailed three-electrode cell setup in an anaerobic CDC biofilm reactor: There are three separate carbon steel coupon working electrodes that can be measured per rod. Each reactor had two rods that were modified for electrochemical analysis, *n* = 3 for both as-received (AR) and polished (P) UNSG10180 carbon steel coupons. Created by BioRender.com.

**Supplementary Table 2.** Environmental conditions on the day the marine sediment was collected on November 19, 2022.

| **Date & Time** | **14/09/2022 07:32** | **14/09/2022 07:47** | **14/09/2022 08:02** | **14/09/2022 08:17** | **14/09/2022 08:32** | **14/09/2022 08:47** | **14/09/2022 09:02** | **14/09/2022 09:17** | **14/09/2022 09:32** |
| --- | --- | --- | --- | --- | --- | --- | --- | --- | --- |
| pH units (pH/ORP) | 8.04 | 8.03 | 8.03 | 8.04 | 8.02 | 8.03 | 8.03 | 8.04 | 8.04 |
| Specific Conductivity mS/cm (Conductivity) | 46.726 | 46.782 | 46.798 | 46.762 | 46.158 | 46.046 | 46.063 | 46.207 | 46.374 |
| Turbidity NTU (Turbidity/Brush) | 3.3 | 1.8 | 2.9 | 1.8 | 1.4 | 2 | 1.7 | 1.7 | 1.9 |
| Barometric Pressure mmHg (Necessary Input) | 765.4 | 765.4 | 765.4 | 765.4 | 765.4 | 765.4 | 765.4 | 765.4 | 765.4 |
| DO mg/L (Hach LDO) | 8.84 | 8.83 | 8.96 | 8.73 | 8.9 | 8.75 | 8.97 | 8.82 | 8.79 |
| Depth meters (Depth 25 m) | 0.127 | 0.124 | 0.125 | 0.117 | 0.125 | 0.116 | 0.112 | 0.116 | 0.112 |
| Turbidity mV (Turbidity/Brush) | 30.198 | 26.851 | 29.206 | 26.79 | 25.966 | 27.288 | 26.568 | 26.641 | 27.012 |
| DO %SAT (Hach LDO) | 97.5 | 97.4 | 99 | 96.4 | 97.4 | 95.7 | 98.1 | 96.7 | 96.6 |
| ORPAgCl mV (pH/ORP) | 205 | 202 | 206 | 208 | 206 | 208 | 210 | 206 | 211 |
| Temperature °C (Temperature) | 11.43 | 11.42 | 11.5 | 11.5 | 11.19 | 11.21 | 11.19 | 11.25 | 11.35 |
| Total Dissolved Solids g/L (Conductivity) | 29.904 | 29.941 | 29.951 | 29.927 | 29.541 | 29.469 | 29.48 | 29.573 | 29.679 |
| Specific Gravity (Depth 25 meter) | 0.03113 | 0.03034 | 0.03009 | 0.02988 | 0.03002 | 0.03006 | 0.03341 | 0.03005 | 0.02979 |
| Density kg/m^3^ (Depth 25 m) | 1.023 | 1.023 | 1.023 | 1.023 | 1.023 | 1.023 | 1.023 | 1.023 | 1.023 |
| Salinity psu (Conductivity) | 1023.22 | 1023.25 | 1023.25 | 1023.23 | 1022.94 | 1022.88 | 1022.89 | 1022.96 | 1023.04 |
| Chlorophyll a µg/L (Chlorophyll a) | 30.53 | 30.57 | 30.58 | 30.55 | 30.12 | 30.03 | 30.05 | 30.15 | 30.27 |

| **Abiotic Coupons** |
| --- |
| 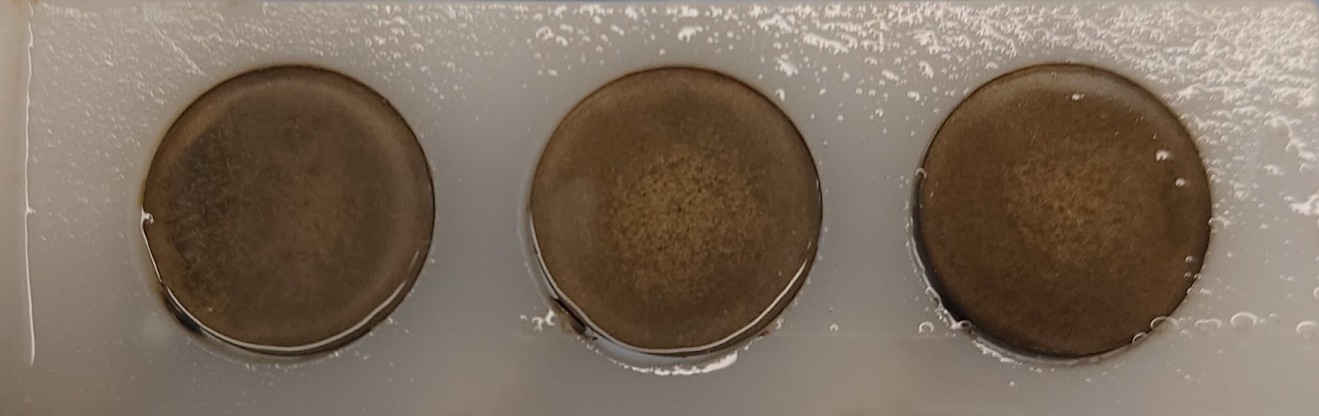 |
| 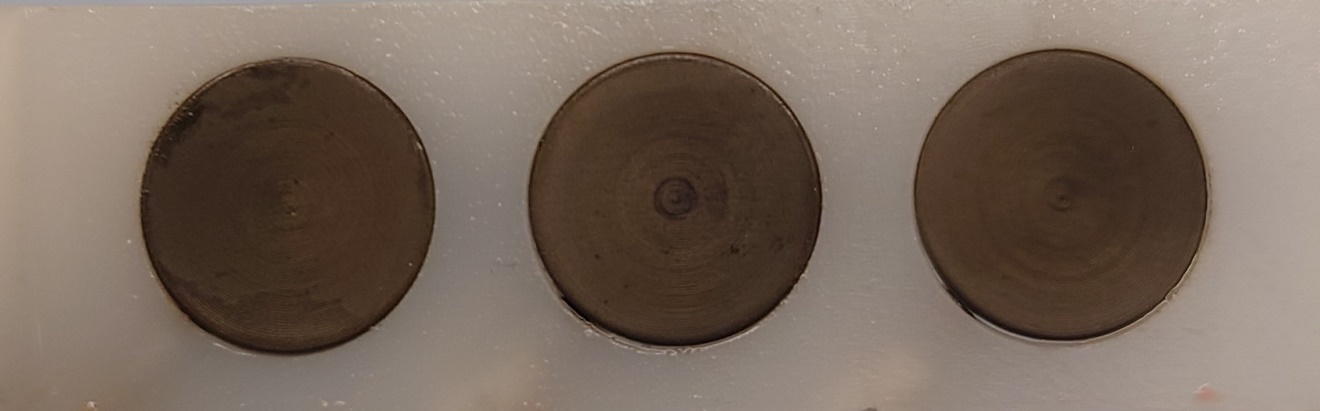 |
| 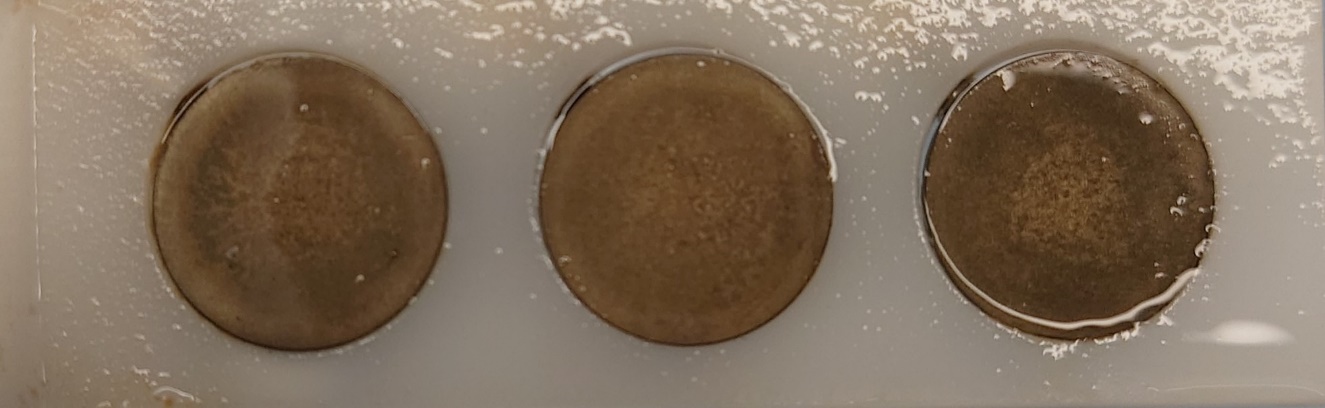 |

**Supplementary Figure 2a.** Photographs taken of the coupon rods taken from the abiotic condition on Day 28, on dismantling the reactor, after exposure to anaerobic MB media for 28 days.

| **Biotic Coupons** |
| --- |
| 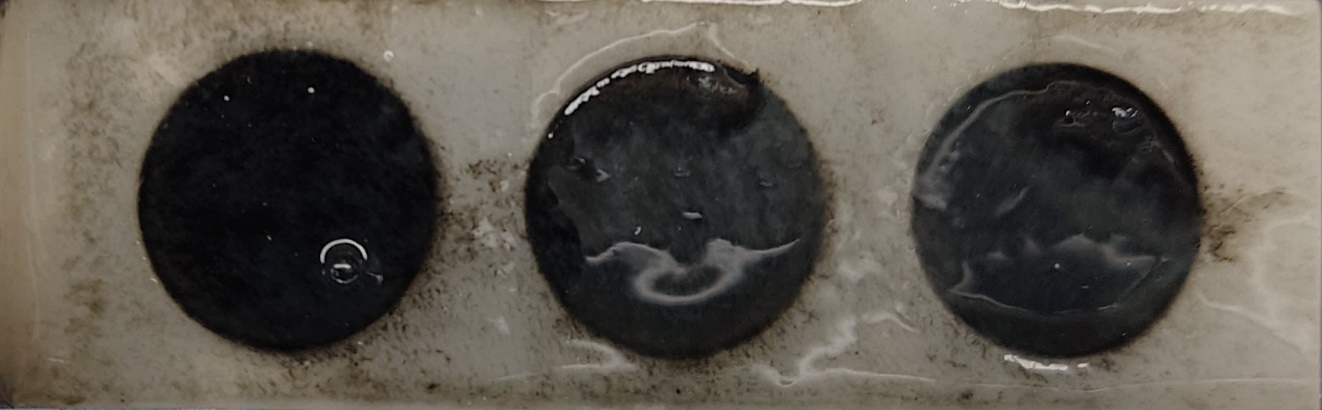 |
| 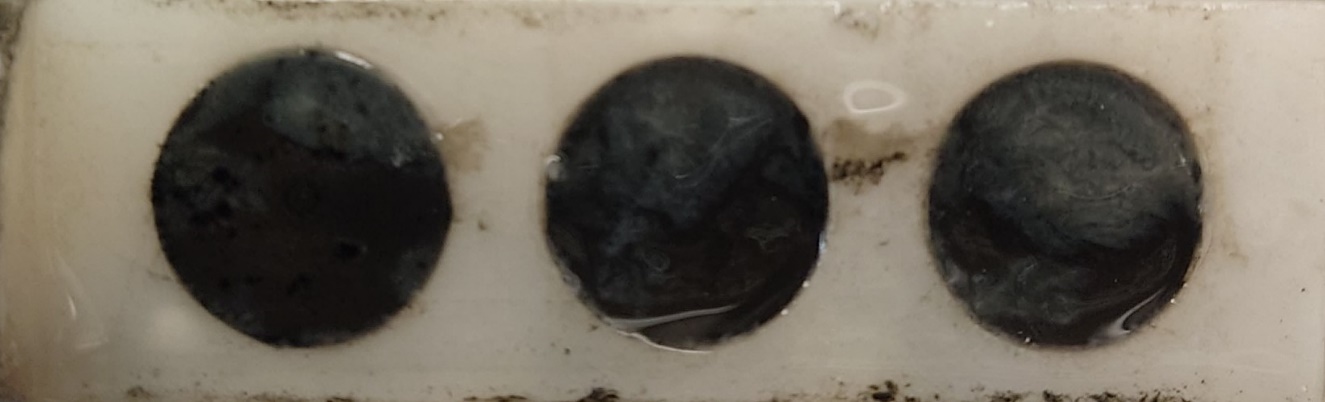 |
| 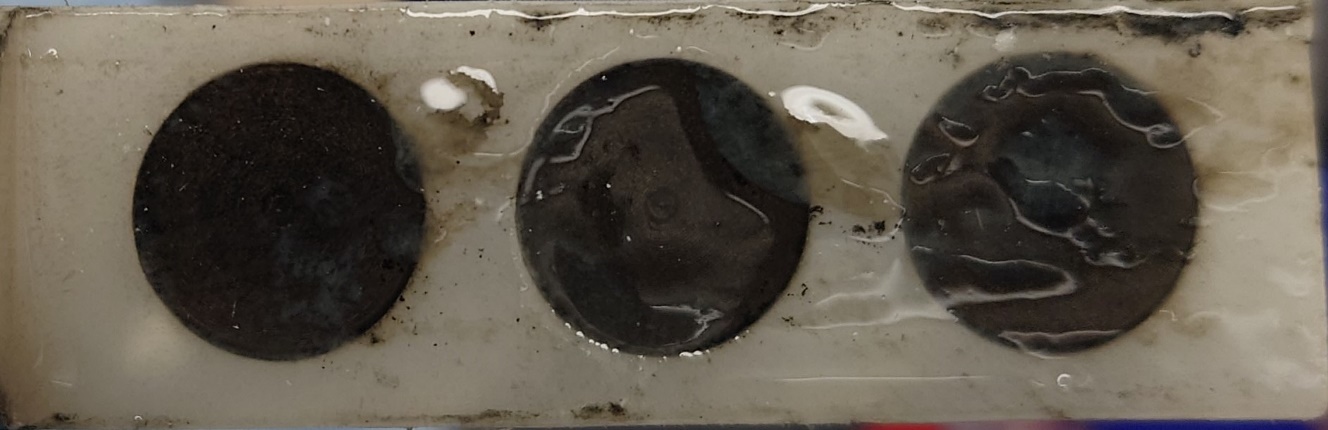 |

**Supplementary Figure 2b.** Photographs taken of the coupon rods taken from the biotic condition on Day 28, on dismantling the reactor, after exposure to anaerobic MB media for 28 days.

| **(a)** **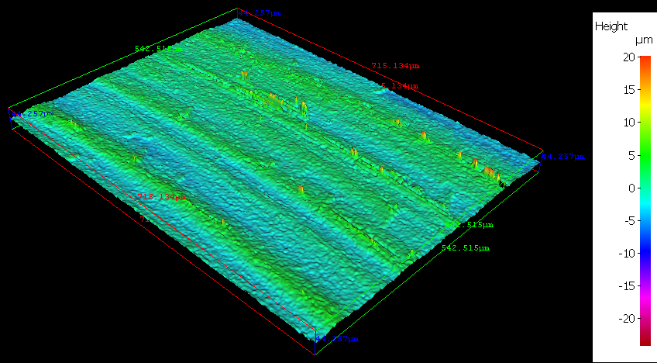**  715 µm  542 µm | **(b)** **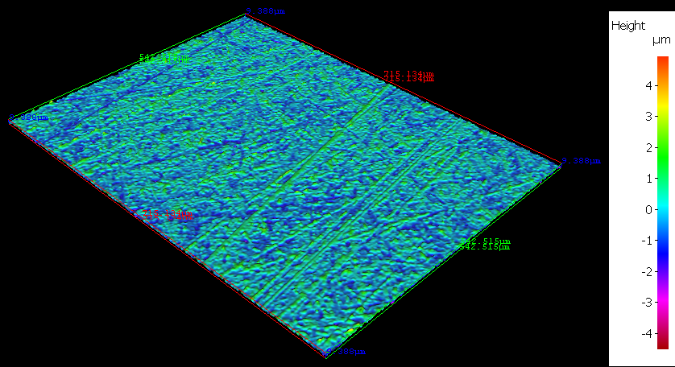**  715 µm  542 µm |
| --- | --- |
| **(c) 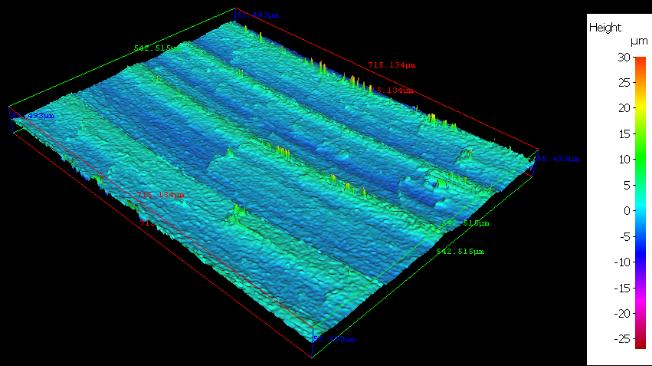**  715 µm  542 µm | **(d) 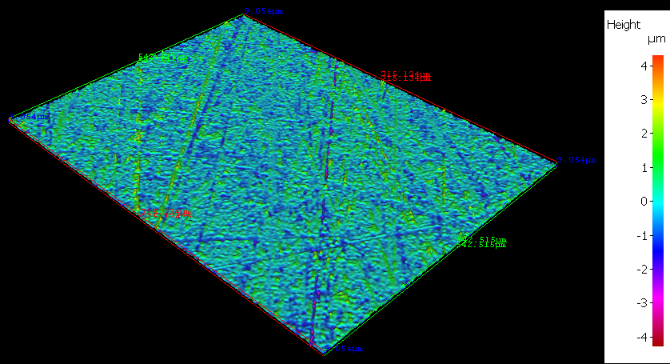**  715 µm  542 µm |

**Supplementary Figure 3.** Three-dimensional optical surface profilometry of UNS G10180 surfaces at day 0. AR coupons for: **(a)** abiotic and **(b)** biotic conditions; and P coupons for: **(c)** abiotic and **(d)** biotic conditions, prior to exposure to anaerobic MB media for 28 days.

**Supplementary Table 3a.** Quantitative surface roughness profiles for AR coupon samples on day 0 and day 28. *R*_a_ average roughness of profile, *R*_t_ maximum peak to valley height of roughness, *R*_z_ mean peak to valley height, *R*_p_ maximum peak height, *R*_v_ maximum valley height, *R*_c_ mean height of profile irregularities, *R*_sm_ mean spacing of profile irregularities, *R*_t_/*R*_z_ extreme scratch/peak value of roughness profile (higher values (>=1) represent larger scratches/peaks).

| **Day** | **Reactor** | ***R*_a_ (µm)** | ***R*_t_ (µm)** | ***R*_z_ (µm)** | ***R*_peak_ (µm)** | ***R*_v_ (µm)** | ***R*_c_ (µm)** | ***R*_sm_ (µm)** | ***R*_t_/*R*_z_ (µm)** |
| --- | --- | --- | --- | --- | --- | --- | --- | --- | --- |
| 0 | Abiotic | 1.2±0.6 | 16.0±10.9 | 9.4±4.8 | 10.6±7.0 | 5.4±5.2 | 8.5±7.7 | 216±190 | 1.6±0.4 |
|  | Biotic | 1.1±0.4 | 13.3±6.4 | 8.2±3.3 | 9.2±5.6 | 4.1±1.8 | 6.3±3.5 | 171±106 | 1.6±0.3 |
| 28 | Abiotic | 1.7±0.8 | 16.7±9.3 | 10.3±3.4 | 7.2±1.9 | 9.5±8.1 | 9.1±6.2 | 163±122 | 1.6±0.5 |
|  | Biotic | 2.0±1.3 | 23.1±13.4 | 14.4±8.1 | 8.6±3.9 | 14.5±10.6 | 10.6±5.6 | 182±80 | 1.6±0.3 |

**Supplementary Table 3b.** Quantitative surface roughness profiles for P coupon samples on day 0 and day 28.

| **Day** | **Reactor** | ***R*_a_ (µm)** | ***R*_t_ (µm)** | ***R*_z_ (µm)** | ***R*_peak_ (µm)** | ***R*_v_ (µm)** | ***R*_c_ (µm)** | ***R*_sm_ (µm)** | ***R*_t_/*R*_z_ (µm)** |
| --- | --- | --- | --- | --- | --- | --- | --- | --- | --- |
| 0 | Abiotic | 0.4±0.05 | 4.0±0.5 | 3.2±0.3 | 2.3±0.4 | 1.7±0.3 | 1.8±0.2 | 34±3 | 1.2±0.1 |
|  | Biotic | 0.4±0.05 | 4.3±0.7 | 3.4±0.4 | 2.5±0.5 | 1.8±0.3 | 1.9±0.2 | 35±5 | 1.3±0.1 |
| 28 | Abiotic | 1.1±0.9 | 21.6±14.4 | 9.5±5.2 | 6.8±4.8 | 14.7±10.4 | 11.1±9.2 | 271±278 | 2.1±0.5 |
|  | Biotic | 2.5±0.6 | 34.6±5.4 | 19.7±3.0 | 10.2±3.2 | 24.4±4.7 | 17.4±2.8 | 251±93 | 1.8±0.3 |

**Supplementary Figure 4.** Equivalent circuit model used to generate EIS parameters shown in Table S7.

| **①** R1+(Q1/Q2+R2)  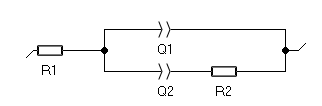 |
| --- |
| **②** R1+(Q1+R2)  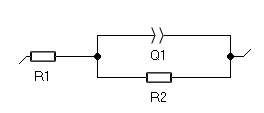 |
| **③** R1+Q1/(R2+Q2/R3)  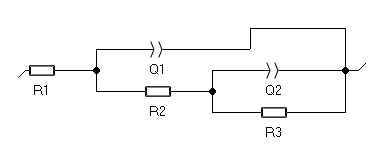 |
| **④** R1+Q1+(Q2/R3)  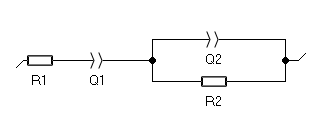 |

**Supplementary Table 4a.** EIS parameters of the carbon steel coupons immersed in anaerobic MB media for the abiotic condition on days 1, 7, 14, 21 and 28. **Equivalent circuit: ①**  – *R*_s_+(*Q*_1_/*Q*_2_+*R*_ct_); **③** – *R*_s_+*Q*_1_/(*R*_film_+*Q*_2_/*R*_ct_).

| **Day** | **Coupon** | ***R*_s_ / W cm^2^** | ***Q*_1_ / mW^–1^ cm^–2^ s*^n^*** | ***n*_1_** | ***R*_film_ / W cm^2^** | ***Q*_2_ / mW^–1^ cm^–2^ s*^n^*** | ***n*_2_** | ***R*_ct_ / W cm^2^** | **c^2^ ×10^-4^** |
| --- | --- | --- | --- | --- | --- | --- | --- | --- | --- |
| 1 | AR **①** | 38.8±3.3 | 0.56±0.07 | 0.78±0.00 | – | 442.3±745.6 | 0.01±0.02 | 415±746 | 15 |
|  | P **①** | 38.9±2.3 | 0.62±0.02 | 0.79±0.01 | – | 97.9±83.3 | 0.00±0.00 | 617±86 | 21 |
| 7 | AR **①** | 17.9±15.6 | 0.61±0.33 | 0.33±0.07 | – | 0.44±0.49 | 0.79±0.29 | 121±82 | 18 |
|  | P **①** | 35.8±1.9 | 0.40±0.09 | 0.51±0.02 | – | 1.33±1.04 | 0.69±0.27 | 184±104 | 25 |
| 14 | AR **③** | 33.6±7.7 | 0.27±0.26 | 0.76±0.28 | 5310±6067 | 0.55±0.34 | 0.89±0.07 | 1007±1680 | 14 |
|  | P **③** | 24.2±8.0 | 0.39±0.22 | 0.40±0.12 | 7.12E+07±6.18E+07 | 0.94±0.94 | 0.74±0.16 | 46±18 | 9.9 |
| 21 | AR **①** | 30.6±4.8 | 0.31±0.03 | 0.43±0.06 | – | 0.25±0.01 | 0.93±0.03 | 117±45 | 18 |
|  | P **①** | 34.0±2.7 | 0.45±0.04 | 0.44±0.03 | – | 1.82±1.45 | 0.73±0.24 | 203±44 | 20 |
| 28 | AR **③** | 14.6±25.3 | 0.29±0.46 | 0.61±0.23 | 1605±1345 | 2.17±2.43 | 0.89±0.09 | 366±561 | 13 |
|  | P **③** | 13.4±14.9 | 0.15±0.26 | 0.57±0.21 | 1935±619 | 0.80±0.16 | 0.83±0.03 | 26±16 | 2.6 |

**Supplementary Table 4b.** EIS parameters of the carbon steel coupons immersed in anaerobic MB media for the biotic condition on days 1, 7, 14, 21 and 28. **Equivalent circuit:** **④**– *R*_s_+*Q*_1_+(*Q*_2_/*R*_ct_).

| **Day** | **Coupon** | ***R*_s_ / W cm^2^** | ***Q*_1_ / mW^–1^ cm^–2^ s*^n^*** | ***n*_1_** | ***Q*_2_ / mW^–1^ cm^–2^ s*^n^*** | ***n*_2_** | ***R*_ct_ / W cm^2^** | **c^2^ ×10^-4^** |
| --- | --- | --- | --- | --- | --- | --- | --- | --- |
| 1 | AR **④** | 41.9±4.7 | 2.56±0.24 | 0.77±0.07 | 0.19±0.02 | 0.90±0.02 | 3565±327 | 28 |
|  | P **④** | 42.4±2.2 | 2.41±0.45 | 0.78±0.08 | 0.19±0.03 | 0.90±0.01 | 2870±844 | 36 |
| 7 | AR **④** | 33.6±3.6 | 1.67±0.48 | 0.88±0.11 | 2.29±0.80 | 0.93±0.08 | 551±429 | 43 |
|  | P **④** | 34.1±2.0 | 1.75±0.42 | 0.88±0.11 | 1.66±0.54 | 0.95±0.07 | 993±915 | 51 |
| 14 | AR **④** | 23.5±20.4 | 2.95±1.18 | 0.90±0.09 | 1.58±2.05 | 0.81±0.18 | 225±353 | 36 |
|  | P **④** | 33.4±1.2 | 2.28±0.19 | 0.88±0.02 | 5.61±9.13 | 0.67±0.58 | 41±69 | 21 |
| 21 | AR **④** | 26.3±23.1 | 2.00±0.16 | 0.84±0.02 | 7.91±6.85 | 1.00±0.00 | 59±29 | 29 |
|  | P **④** | 12.0±20.7 | 1.86±0.08 | 0.88±0.01 | 3.76±6.51 | 0.62±0.34 | 61±44 | 10 |
| 28 | AR **④** | 37.0±3.2 | 1.93±0.17 | 0.84±0.02 | 6.51±5.53 | 1.00±0.00 | 59±60 | 44 |
|  | P **④** | 32.6±1.2 | 2.26±0.87 | 0.93±0.07 | 1.16±1.41 | 0.94±0.10 | 457±788 | 26 |

| 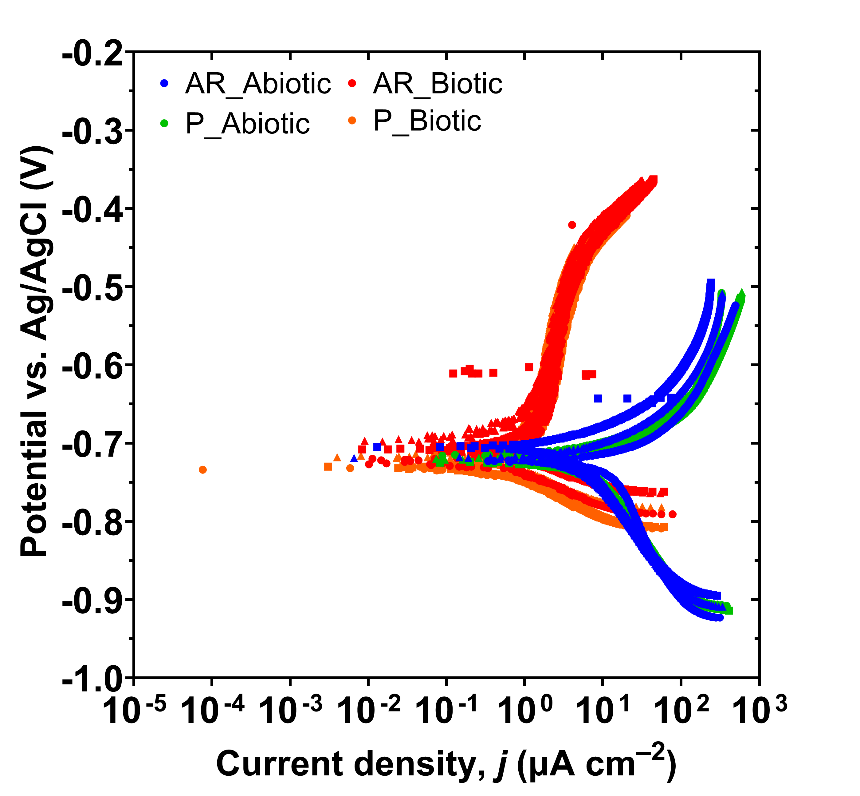  **Supplementary Figure 5.** Potentiodynamic polarisation curves for the abiotic and biotic AR and P, UNS G10180 carbon steel coupons, at ambient temperature after exposure to anaerobic MB media for 28 days. Scan rate of 0.5 mV s^–1^ and reactor stirrer at 50 rpm. Dissolved oxygen levels were 0.5 ppm (abiotic) and 0.0 ppm (biotic), at Day 28. |
| --- |

**Supplementary Table 5.** Fitted electrochemical parameters from polarisation curves; comparison between the abiotic and biotic AR and P UNS G10180 carbon steel coupons after exposure to anaerobic MB media for 28 days.

|  | **Coupon** | ***j*_corr_ / A cm ^– 2^** | ***E*_corr_ *vs*. Ag/AgCl / V** | ***β_a_* (mV dec^-1^)** | ***β_c_* (mV dec^-1^)** |
| --- | --- | --- | --- | --- | --- |
| **Abiotic** | AR | 22.4 ± 20.1 | -727 ± 3 | 157 ± 52 | 268 ± 67 |
|  | P | 15.7 ± 2.49 | -720 ± 4 | 125 ± 12 | 266 ±39 |
| **Biotic** | AR | 0.44 ± 0.22 | -710 ± 15 | 213 ± 52 | 38 ±11 |
|  | P | 0.70 ± 0.33 | -727 ± 7 | 276 ± 54 | 54 ± 15 |

| 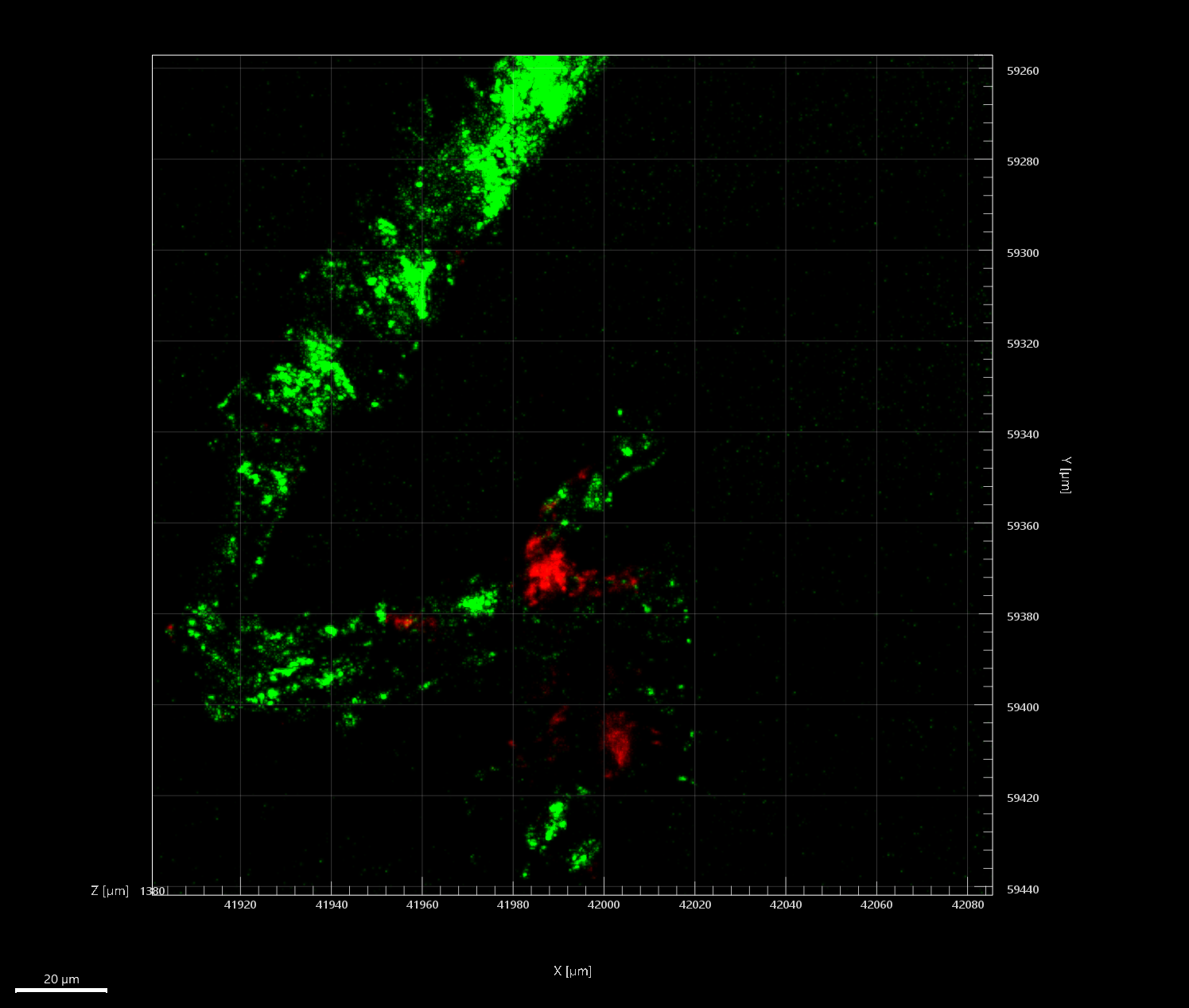 | 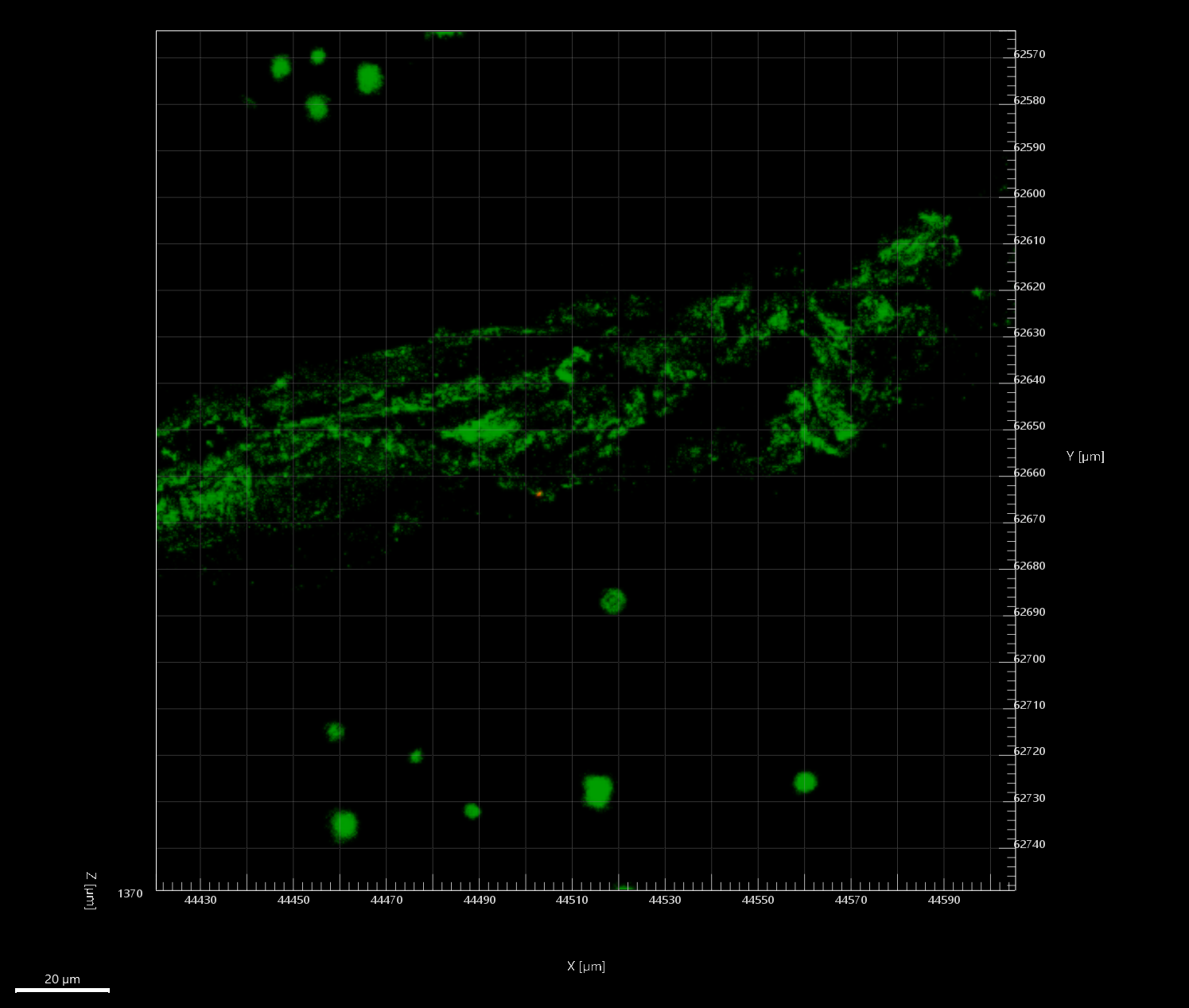 |
| --- | --- |

**Supplementary Figure 6.** Confocal images of biofilms formed over UNS G10180 carbon steel surfaces for **(a)** AR and **(b)** P biofilms, after exposure to anaerobic MB media for 28 days.

**Supplementary Table 6.** List of top 25 microbial genera identified through 16S rRNA amplicon sequencing with two target region, V3-4 for bacteria and archaea, after exposure to anaerobic MB media for 28 days.

| **Genus** | **Sediment** | **Day0** | **Day28** | **AR** | **25M** |
| --- | --- | --- | --- | --- | --- |
| *Desulfovibrio* | 0.08945 | 0.09537 | 32.02 | 54.73 | 52.49 |
| *Clostridium* | 0.1979 | 13.26 | 0.7714 | 3.671 | 3.613 |
| *Klebsiella* | 0.09221 | 11.14 | 3.758 | 2.412 | 2.002 |
| *Sulfurovum* | 9.308 | 0.05396 | 0.001624 | 0.000427 | 0.000813 |
| *Escherichia* | 0.3072 | 8.908 | 4.851 | 3.425 | 3.041 |
| *Parabacteroides* | 0.004009 | 7.908 | 6.417 | 3.84 | 4.74 |
| *Veillonella* | 0.000251 | 7.777 | 0.04258 | 0.1269 | 0.2159 |
| *Anaerotignum* | 0.000752 | 7.061 | 3.891 | 1.256 | 1.512 |
| *Bacteroides* | 0.004009 | 6.807 | 4.553 | 2.663 | 2.405 |
| *Sulfurospirillum* | 0.01879 | 0.000267 | 6.502 | 0.4396 | 0.5303 |
| *Salmonella* | 0.1947 | 5.843 | 5.63 | 2.26 | 2.059 |
| *Desulfuromonas* | 5.616 | 0.02698 | NA | NA | NA |
| *Enterobacter* | 0.01077 | 2.699 | 5.362 | 1.23 | 1.121 |
| *Candidatus Prometheoarchaeum* | 5.229 | 0.02351 | NA | NA | NA |
| *Anaerotruncus* | 0.01077 | 0.276 | 5.202 | 1.447 | 1.483 |
| *Terrisporobacter* | 0.003508 | 5.164 | 0.007398 | 0.349 | 0.3042 |
| *Desulfosarcina* | 4.827 | 0.02244 | NA | NA | NA |
| *Thiohalobacter* | 3.976 | 0.01924 | NA | 0.000854 | 0.000407 |
| *Candidatus Methanoplasma* | 3.699 | NA | NA | NA | NA |
| *Kineobactrum* | 3.526 | 0.01229 | NA | NA | NA |
| *Fusobacterium* | 0.002004 | 1.875 | 1.166 | 2.857 | 3.313 |
| *Providencia* | 0.000251 | 0.05183 | 3.259 | 0.5887 | 0.501 |
| *Ruthenibacterium* | 0.006515 | 2.806 | 2.349 | 1.753 | 2.235 |
| *Aliarcobacter* | 0.01278 | 0.000802 | 2.759 | 0.9953 | 0.9821 |
| *Wenzhouxiangella* | 2.629 | 0.01015 | NA | NA | NA |

| 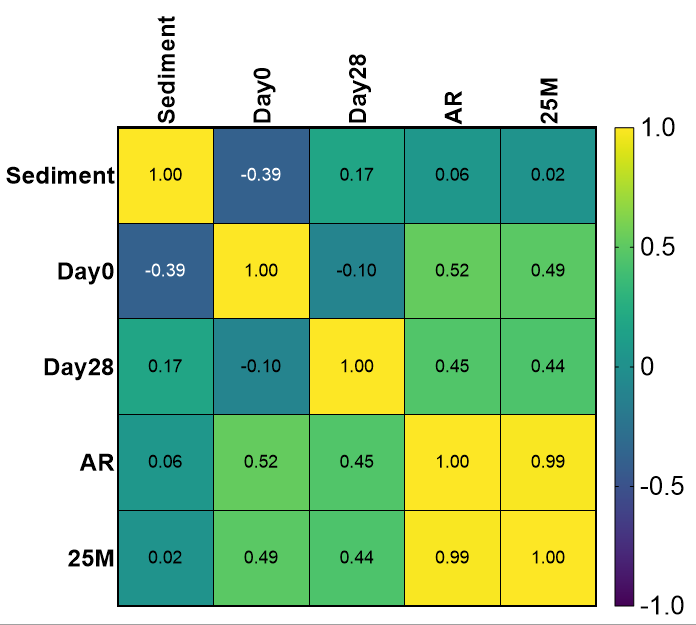 |
| --- |

**Supplementary Figure 7.** Spearman correlation coefficients for environmental marine sediment, Day 0, and Day 28 planktonic samples, AR and P biofilms, after exposure to anaerobic MB media for 28 days.

| **(a)**  **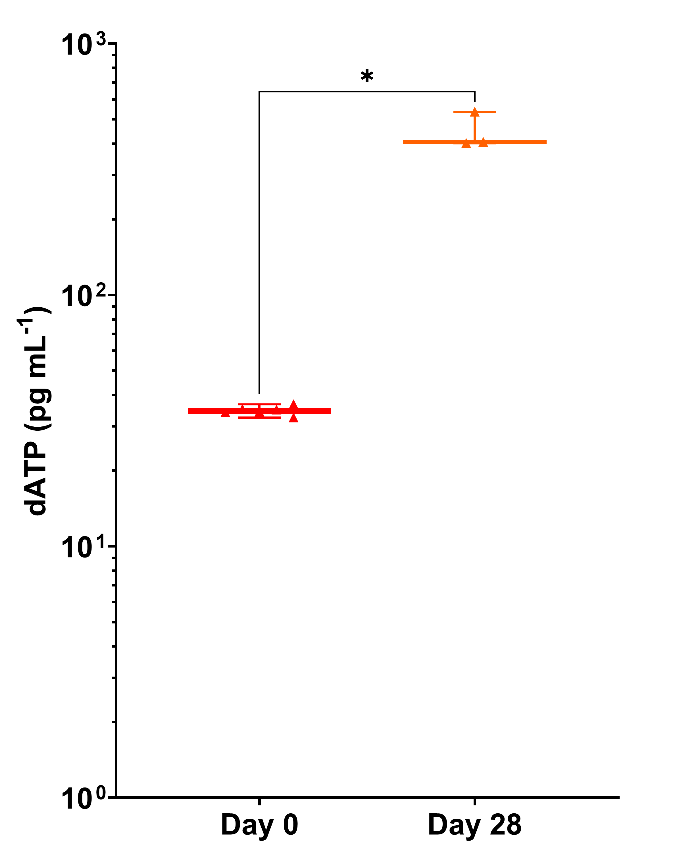** | **(b)**  **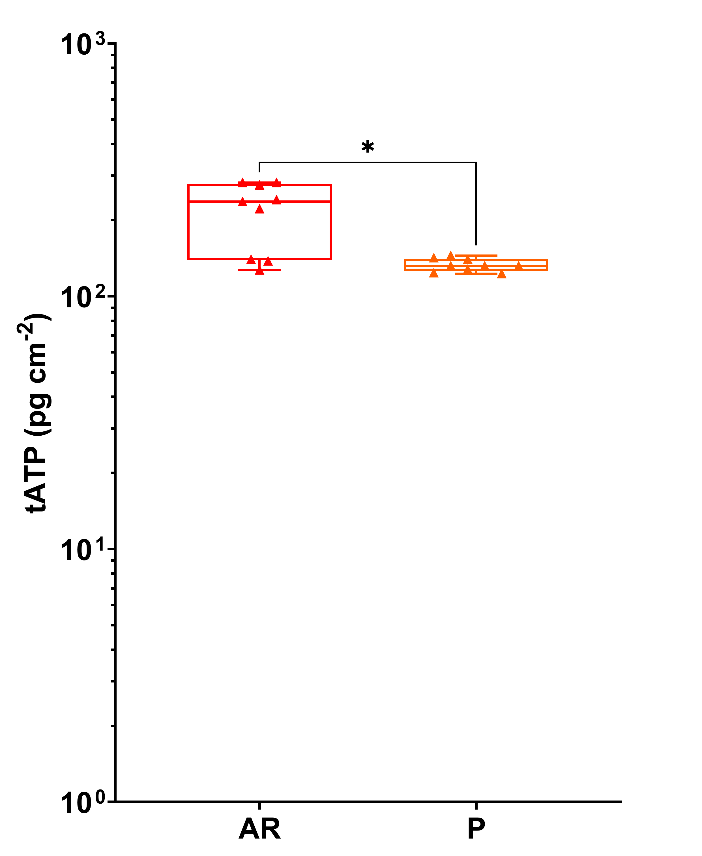** |
| --- | --- |

**Supplementary Figure 8.** (a) Dissolved ATP (dATP) concentrations comparing the anaerobic MB media taken on Day 0 and Day 28 (P < 0.05) and (b) Total ATP (tATP) concentration comparing the biofilm of the AR and P coupons (P < 0.05), from the biotic condition, after exposure to anaerobic MB media for 28 days.
